# Main Determinants Affecting the Antiproliferative Activity of Stilbenes and Their Gut Microbiota Metabolites in Colon Cancer Cells: A Structure–Activity Relationship Study

**DOI:** 10.3390/ijms232315102

**Published:** 2022-12-01

**Authors:** Antonio González-Sarrías, Juan Carlos Espín-Aguilar, Salvador Romero-Reyes, Julio Puigcerver, Mateo Alajarín, José Berná, María Victoria Selma, Juan Carlos Espín

**Affiliations:** 1 Laboratory of Food and Health, Research Group on Quality, Safety, and Bioactivity of Plant Foods, Department of Food Science and Technology, CEBAS-CSIC, Campus de Espinardo, P.O. Box 164, 30100 Murcia, Spain; 2 Department of Organic Chemistry, Faculty of Chemistry, University of Murcia, 30100 Murcia, Spain

**Keywords:** resveratrol, dihydroresveratrol, lunularin, 4-hydroxydibenzyl, stilbenes, gut microbiota, metabotype, colon cancer, Caco-2, HT-29, CCD18-Co

## Abstract

*trans*-Resveratrol can be catabolized by the gut microbiota to dihydroresveratrol, 3,4′-dihydroxy-trans-stilbene, lunularin, and 4-hydroxydibenzyl. These metabolites can reach relevant concentrations in the colon. However, not all individuals metabolize RSV equally, as it depends on their RSV gut microbiota metabotype (i.e., lunularin producers vs. non-producers). However, how this microbial metabolism affects the cancer chemopreventive activity of stilbenes and their microbial metabolites is poorly known. We investigated the structure–antiproliferative activity relationship of dietary stilbenes, their gut microbial metabolites, and various analogs in human cancer (Caco-2 and HT-29) and non-tumorigenic (CCD18-Co) colon cells. The antiproliferative IC_50_ values of pterostilbene, oxy-resveratrol, piceatannol, resveratrol, dihydroresveratrol, lunularin, 3,4′-dihydroxy-*trans*-stilbene, pinosylvin, dihydropinosylvin, 4-hydroxy-*trans*-stilbene, 4-hydroxydibenzyl, 3-hydroxydibenzyl, and 4-*trans*-stilbenemethanol were calculated. IC_50_ values were correlated with 34 molecular characteristics by bi- and multivariate analysis. Little or no activity on CCD18-Co was observed, while Caco-2 was more sensitive than HT-29, which was explained by their different capacities to metabolize the compounds. Caco-2 IC_50_ values ranged from 11.4 ± 10.1 μM (4-hydroxy-*trans*-stilbene) to 73.9 ± 13.8 μM (dihydropinosylvin). In HT-29, the values ranged from 24.4 ± 11.3 μM (4-hydroxy-*trans*-stilbene) to 96.7 ± 6.7 μM (4-hydroxydibenzyl). At their IC_50_, most compounds induced apoptosis and arrested the cell cycle at the S phase, pterostilbene at G_2_/M, while 4-hydroxy-*trans*-stilbene and 3,4′-dihydroxy-*trans*-stilbene arrested at both phases. Higher Connolly values (larger size) hindered the antiproliferative activity, while a lower pKa1 enhanced the activity in Caco-2, and higher LogP values (more hydrophobicity) increased the activity in HT-29. Reducing the styrene double bond in stilbenes was the most critical feature in decreasing the antiproliferative activity. These results (i) suggest that gut microbiota metabolism determines the antiproliferative effects of dietary stilbenes. Therefore, RSV consumption might exert different effects in individuals depending on their gut microbiota metabotypes associated with RSV metabolism, and (ii) could help design customized drugs with a stilbenoid and (or) dibenzyl core against colorectal cancer.

## 1. Introduction

Resveratrol (*trans*-resveratrol, RSV) is the most relevant dietary stilbene, primarily recognized by its vascular protective effects [1,2,3,4]. In addition, many animal studies also support the anticancer effects of RSV, including against colorectal cancer, although the human evidence is still scant [5,6]. Nevertheless, not all human trials describe measurable RSV effects [7,8], and there is also controversy regarding the effective RSV concentration since more is not always better [9,10].

A relevant factor contributing to the substantial human inter-individual variability in (poly)phenol health effects is their differential metabolism by the gut microbiota [11]. Most (poly)phenols are catabolized by the individuals’ gut microbiota to a different extent, yielding a gradient of microbial-derived metabolite production (i.e., the so-called high vs. low producers) [11,12]. In addition to the higher or lower capacity of producing metabolites, some specific metabolites are only produced by some gut microbial ecologies, yielding the so-called gut microbiota metabotypes associated with (poly)phenols metabolism [12]. Well-known metabotypes are those related to the metabolism of isoflavones (equol producers vs. non-producers) [13] and ellagic acid (urolithin metabotypes A, B, and 0) [14]. Recently, a novel metabotype associated with RSV metabolism has been identified, i.e., lunularin (LUNU) producers vs. non-producers, with a gradient of LUNU production within LUNU producers [15]. In healthy volunteers, RSV is reduced by the gut microbiota to dihydroresveratrol (DHRSV), which only in the LUNU-producer metabotype is dehydroxylated at the 5-position to yield LUNU, and then, LUNU is further dehydroxylated at the 3-position to produce 4-hydroxydibenzyl (4HDB) [15,16]. Therefore, as in the case of other (poly)phenols, different gut microbial ecologies confer high inter-individual variability, affecting the outcome of studies with RSV since the biological activity of the derived microbial metabolites might differ from that of RSV [11,12,15,17,18].

Screening in vitro studies to test potential biological activities, including anticancer, should be based on the assay of plausible physiological concentrations that can be attained in vivo [9,19]. In this regard, RSV, and especially its gut microbial-derived metabolites, such as DHRSV or LUNU, can reach the human colon at relevant concentrations (>100 μM) [15]. However, while the anticancer activity of RSV has been widely reported, little or nothing is known about this activity for its physiologically relevant microbial metabolites. In addition, no previous studies have identified the main structural determinants affecting the antiproliferative activity of stilbenes and their physiologically relevant gut microbial-derived dibenzyls.

Taking into account the recently described RSV metabolism by the gut microbiota [15], we hypothesized that the styrene double bond reduction and sequential dehydroxylation of RSV by the gut microbiota could affect the antiproliferative activity of RSV against human colon cancer cells. Thus, we aimed here to investigate, through a structure–activity relationship study, the primary molecular determinants of dietary stilbenes, their gut microbial-derived metabolites, and other dietary and synthetic analogs concerning their antiproliferative capacity and some associated mechanisms in different colon cancer cells.

## 2. Results

### 2.1. Antiproliferative Activity of Stilbenes and Dibenzyls

**Figure 1:**
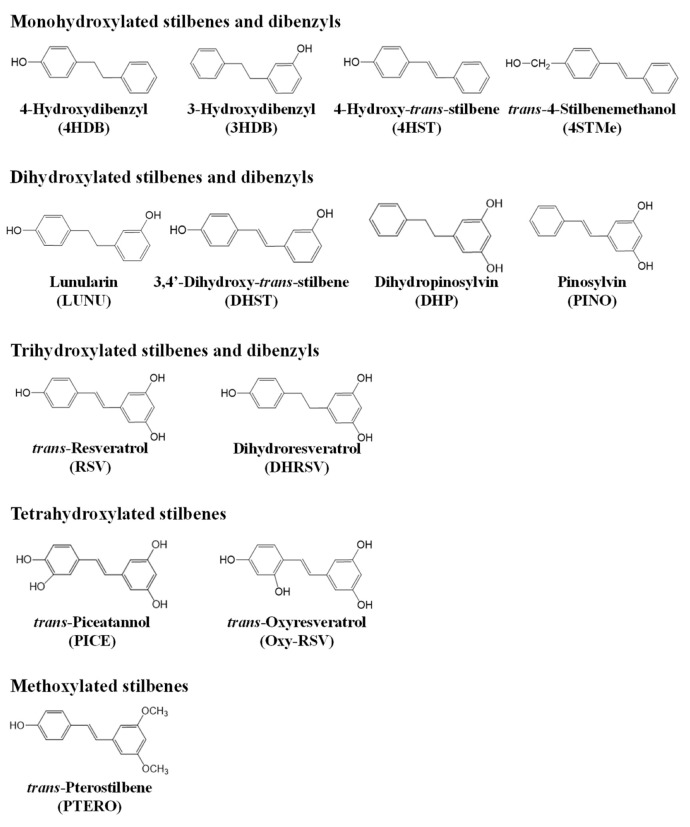
Structures of the stilbenes and dibenzyls that were assayed in this study.

Figure 1 shows the stilbenes and dibenzyls assayed in the present study.

**Figure 2:**
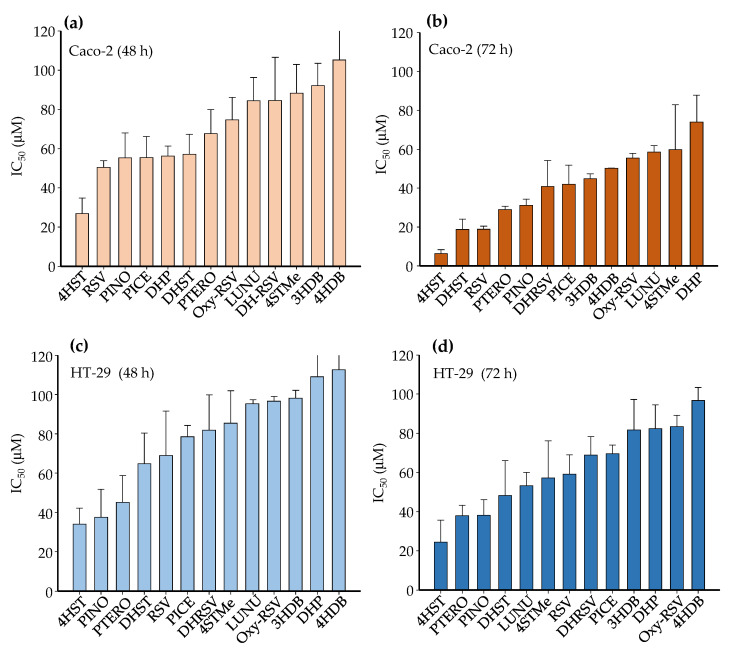
Antiproliferative IC_50_ values of stilbenes and dibenzyls in Caco-2 (**a**,**b**) and HT-29 (**c**,**d**) cells at 48 and 72 h. Values are shown as mean ± SD (n = 3).

The stilbene 4HST showed the highest antiproliferative activity (the lowest IC_50_ values) in Caco-2 and HT-29 cells (Table 1, Figure 2). IC_50_ values were lower for Caco-2 than HT-29, suggesting that HT-29 cells were less sensitive than Caco-2 cells to these molecules. Although IC_50_ values decreased with incubation time in both cell lines, IC_50_ reduction was more evident in Caco-2 than in HT-29 (Figure S1). In Caco-2, IC_50_ values decreased from 1.25-fold (DHRSV) to 5-fold (4HST) after incubation from 48 to 72 h (mean decrease of 2-fold) (Figure S1). In contrast, the mean IC_50_ decrease was 1.25-fold in HT-29 from 48 to 72 h, ranging from no change (PINO) to a 1.4-fold reduction (4HST) (Figure S1).

Regarding the non-tumorigenic CCD18-Co cell line, the antiproliferative effects of stilbenes and dibenzyls could not be determined or were much lower (higher IC_50_ values) than in Caco-2 and HT-29 cells (Figure S2). In this case, in contrast to cancer cells, DHRSV and LUNU were the most active compounds at 72 h (IC_50_ values of 53 μM and 67 μM, respectively).

### 2.2. Cell Metabolism of Stilbenes and Dibenzyls

**Figure 3:**
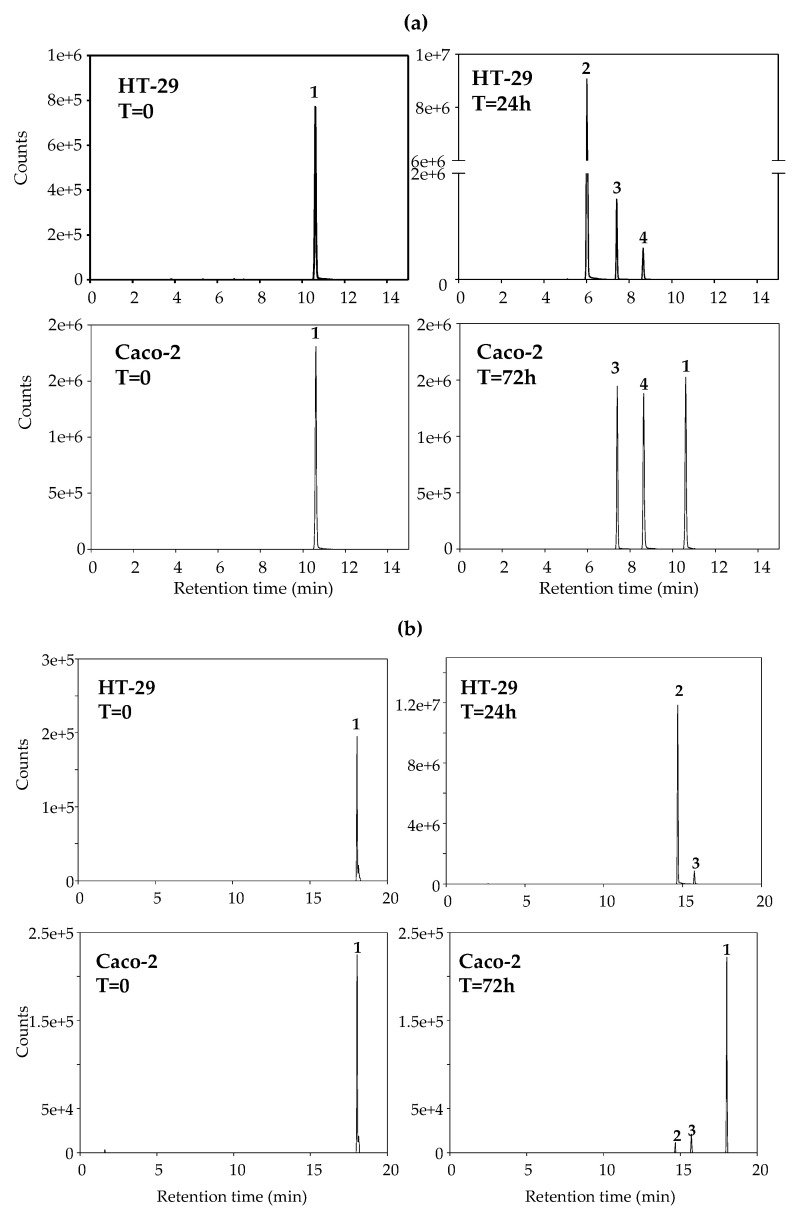
Extracted ion chromatograms showing the phase-II metabolism of (RSV) (**a**) and 4-hydroxy-*trans*-stilbene (4HST) (**b**) by HT-29 (0 and 24 h) and Caco-2 cells (0 and 72 h). (**a**): RSV (**1**, *m*/*z^−^* 227.0714), RSV 4′-*O*-glucuronide (**2**, *m*/*z^−^* 403.1035), RSV 3-*O*-glucuronide (**3**, *m*/*z^−^* 403.1035), and RSV 3-*O*-sulfate (**4**, *m*/*z^−^* 307.0282); (**b**): 4HST (**1**, *m*/*z^−^* 195.0815), 4HST-*O*-glucuronide (**2**, *m*/*z^−^* 371.1136), and 4HST-*O*-sulfate (**3**, *m*/*z^−^* 275.0384).

**Figure 4:**
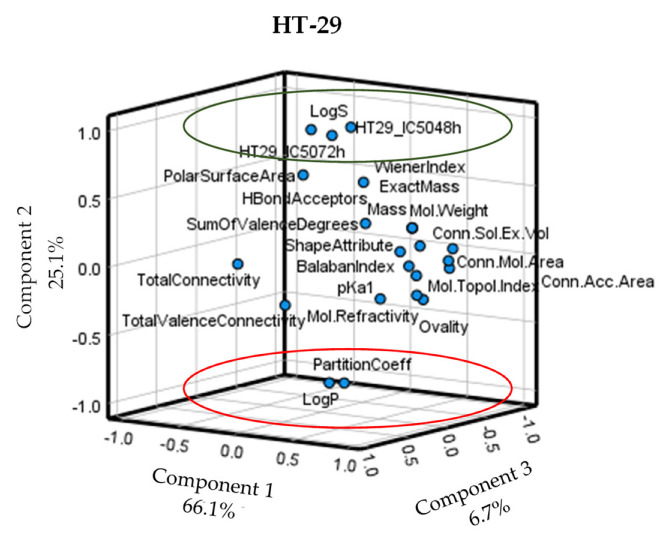
Principal component analysis in a three-dimensional rotated space for HT-29 cells. Red circle, significant inverse variables vs. IC_50_ values; green circle, significant direct variables vs. IC_50_ values.

We next evaluated the phase-II metabolism of some representative stilbenes and dibenzyls in Caco-2 and HT-29 to explore whether the different abilities of these cell lines to metabolize the compounds could explain, at least partially, why IC_50_ values decreased less in HT29 than in Caco-2 upon incubation time. Notably, HT-29 cells metabolized the compounds more extensively and rapidly than Caco-2 cells. Figure 3 shows the phase-II metabolism of RSV (Figure 3a) and 4HST (Figure 3b), and Figure S3 shows the metabolism of DHRSV (a) and PTERO (b).

While 100% RSV was metabolized by HT-29 cells after 24 h, only 14% RSV was metabolized by Caco-2 cells after 72 h (Figure 3a). Of note, HT-29 cells primarily glucuronidated RSV in the 4′-position, yielding RSV 4′-*O*-glucuronide. In contrast, this metabolite was not produced by Caco-2 cells (Figure 3a). Like RSV, 4HST was fully metabolized by HT-29 cells after 24 h, primarily to 4HST-glucuronide, while Caco-2 cells hardly metabolized 4HST after 72 h (Figure 3b). The same metabolic patterns and kinetics were also observed in the case of PTERO and DHRSV (Figure S3), although DHRSV was the only compound fully metabolized by Caco-2 cells after 72 h.

### 2.3. Structure–Antiproliferative Activity Analysis of Stilbenes and Dibenzyls in Caco-2 and HT-29 Cells

Next, we explored which structural determinants of the molecules were more or less associated with the observed antiproliferative IC_50_ values. The Supporting Information includes the approximately 30 molecular characteristics and structurally related indexes studied for all the compounds (Figure S4). Among others, these determinants include those related to the size and shape of the molecule (Connolly’s values, ovality, surface area, etc.), ionization (pKa values), and solubility (LogP, LogS, etc.). The principal component analysis in a three-dimensional rotated space showed which possible variables related to the molecular topology and the physical-chemical properties of the compounds were closer to the antiproliferative IC_50_ values. In the Caco-2 cell line, no relationship between the features of the compounds and the IC_50_ values became significant, which suggested the antiproliferative activity of stilbenes and dibenzyls did not depend on any specific and single structural determinant in Caco-2 cells (Figure S5). Still, the rotated component matrix revealed values close to statistical significance, i.e., pKa1 and Connolly’s solvent-excluded volume, which became potential candidates for further bivariate correlations with the IC_50_ values (Figure S5). In the case of HT-29, the same analysis showed direct (green) or inverse (red) significant associations in component 2 for several characteristics vs. IC_50_ values. The most evident inverse correlation was observed for LogP values (the higher LogP, the lower IC_50_, and, thus, more activity), and a direct correlation (the higher value, the lower activity) mainly for LogS (Figure 4). Therefore, these characteristics were selected for further bivariate correlations with IC_50_ values.

**Figure 5:**
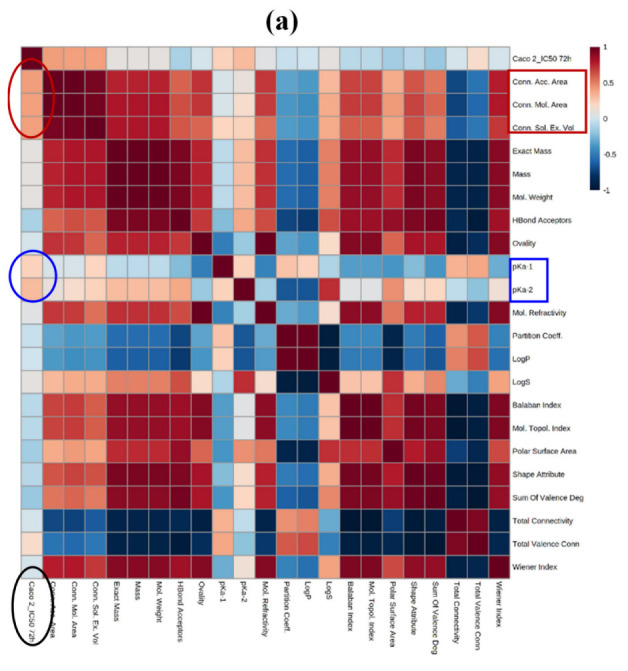

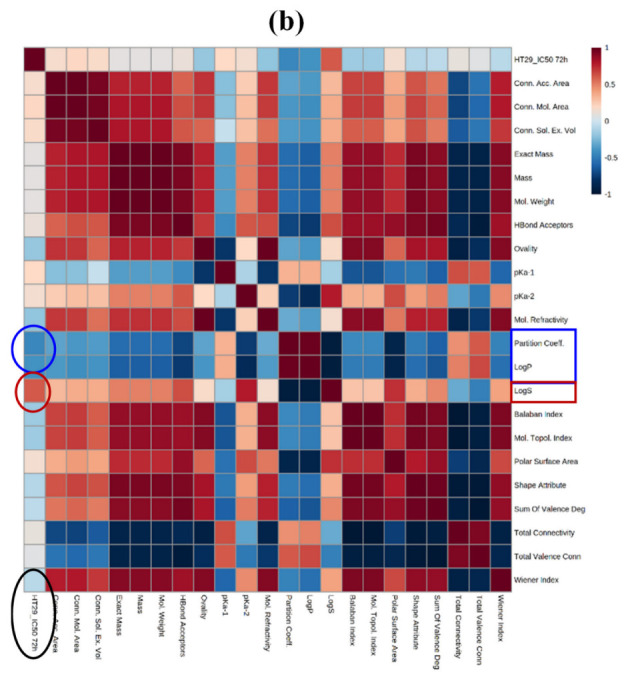
Heat map showing the correlations (Spearman) between the main variables considered and the IC_50_ values after 72 h for Caco-2 (**a**) and HT-29 (**b**) cells. Darker red means a higher direct correlation, and darker blue indicates a higher inverse correlation.

Then, we complemented the three-dimensional rotated analyses with a heat map to check all the associations between the different variables considered and the IC_50_ values for the two cell lines. In Caco-2 cells, a direct association was observed between IC_50_ and Connolly’s areas and volume, while the association was inverse between IC_50_ and pKa1 and pKa2 (Figure 5a). Regarding HT-29 cells, the association was inverse between IC_50_ and LogS, and direct between IC_50_ and LogP, and the partition coefficient, all features related to the solubility of the molecules (Figure 5b).

**Figure 6:**
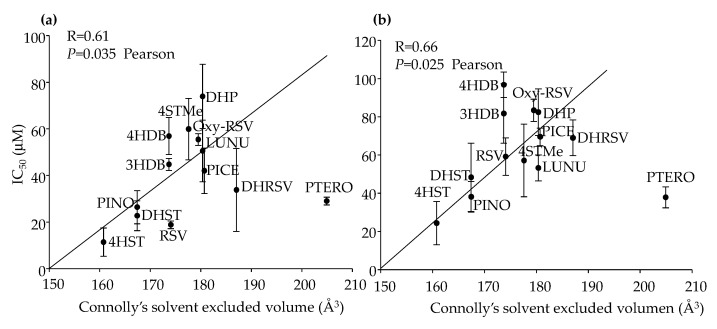
IC_50_ dependence on Connolly’s solvent-excluded volume in Caco-2 (**a**) and HT-29 (**b**) cells. Pearson correlation values were calculated excluding PTERO. Values are shown as mean ± SD (n = 3).

Once we obtained potential features correlated with IC_50_ values, we evaluated specific bivariate correlations. For example, in the case of Connolly’s solvent-excluded volume, we observed a proportional increase in IC_50_ (less activity of the compounds) in both lines with the increase in area (Figure 6). Thus, this characteristic seems to have an evident influence on IC_50_ values. However, it became significant only when PTERO was excluded from the correlation analysis since PTERO did not follow the observed trend, suggesting that additional determinants influenced IC_50_ values. In addition, Connolly’s molecular area and Connolly’s accessible area, proportional features to Connolly’s solvent-excluded volume, showed similar trends (results not shown).

**Figure 7:**
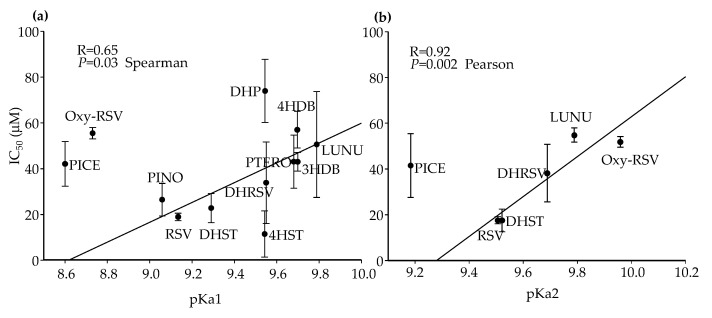
IC_50_ values dependence on pKa1 (**a**) and pKa2 (**b**) in Caco-2 cells after 72 h. Spearman and Pearson correlation values were calculated excluding PICE and Oxy-RSV (**a**) and PICE (**b**). Values are shown as mean ± SD (n = 3).

Regarding pKa values, a significant trend between IC_50_ and pKa1 values was observed in Caco-2 cells but only when Oxy-RSV and PTERO were excluded from the correlation analysis, which did not follow the same trend (Figure 7a). In the case of pKa2 values, the same tendency was observed for those compounds that presented two or more hydroxyl groups (Figure 7b). Similarly, the analysis became significant when PICE was excluded from the correlation. Thus, other structural determinants influenced IC_50_ values in Caco-2 cells. In the case of HT-29, pKa and IC_50_ values also showed the same trend, but it was weaker than for Caco-2 cells (results not shown).

**Figure 8:**
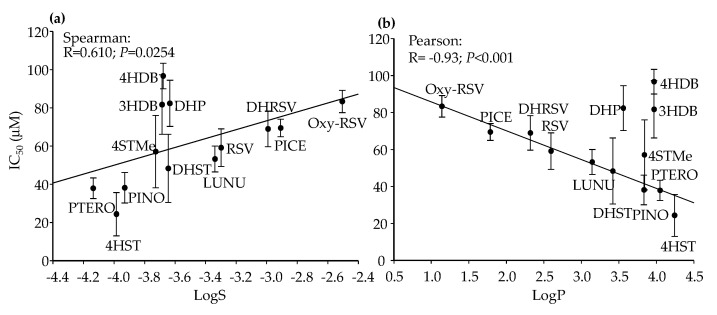
IC_50_ values dependence on LogS (**a**) and LogP (**b**) in HT-29 cells after 72 h. Pearson correlation values were calculated excluding DHP, 3HDB, and 4HDB in (**b**). Values are shown as mean ± SD (n = 3).

In HT-29 cells, a significant direct correlation between IC_50_ and LogS was found (Figure 8a). However, as expected, since LogS is opposite to LogP, this association was inverse but only became significant for LogP when DHP, 4HDB, and 3HDB were excluded from the analysis (Figure 8b). Therefore, a higher hydrophobicity (lower LogS, higher LogP) was associated with more activity (lower IC_50_ values) in HT-29 cells. As in the previous bivariate associations, some compounds did not follow the trend, which reinforced the existence of an interaction of several structural characteristics on the IC_50_ values.

### 2.4. Effect of Stilbenes and Dibenzyls on Cell Cycle and Apoptosis Induction in Caco-2 Cells

**Figure 9:**
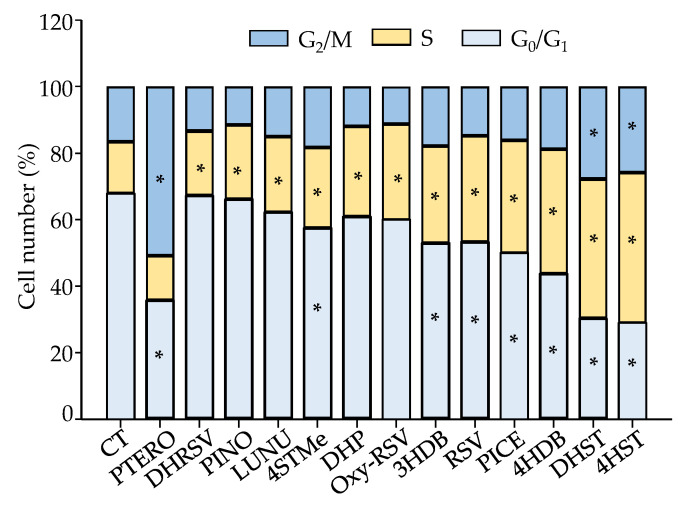
Cell cycle distribution in Caco-2 cells after 72 h. IC_50_ concentrations (Table 1) were assayed for each compound. Data are shown as the mean ± SD of 3 independent experiments. * *p* < 0.05 vs. control (CT).

The cell cycle distribution and apoptosis induction were explored to delve into some of the mechanisms involved in the antiproliferative action exerted by these compounds. We evaluated the effects in the Caco-2 cell line using the corresponding IC_50_ concentration for each compound at 72 h of treatment. After 72 h, the percentage of control cells in the G_0_/G_1_ phase was 67.6 ± 1.8%, while cells in S and G_2_/M phases were 15.5 ± 5.3% and 16.1 ± 3.7%, respectively. However, compared to control cells, most compounds exerted a significant increase at the S phase (from 8 to 30%, depending on the metabolite; *p* < 0.05) (Figure 9). This increase was accompanied, in most cases, by a significant (*p* < 0.05) decrease in cells at G_0_/G_1_ phase. The only differences were observed for PICE, 4HST, and DHST. After PTERO treatment, a significant increase (*p* < 0.05) was observed only at the G_2_/M phase. However, in the case of 4HST and DHST, a significant increase (*p* < 0.05) was observed at both phases, S and G_2_/M (Figure 9).

**Figure 10:**
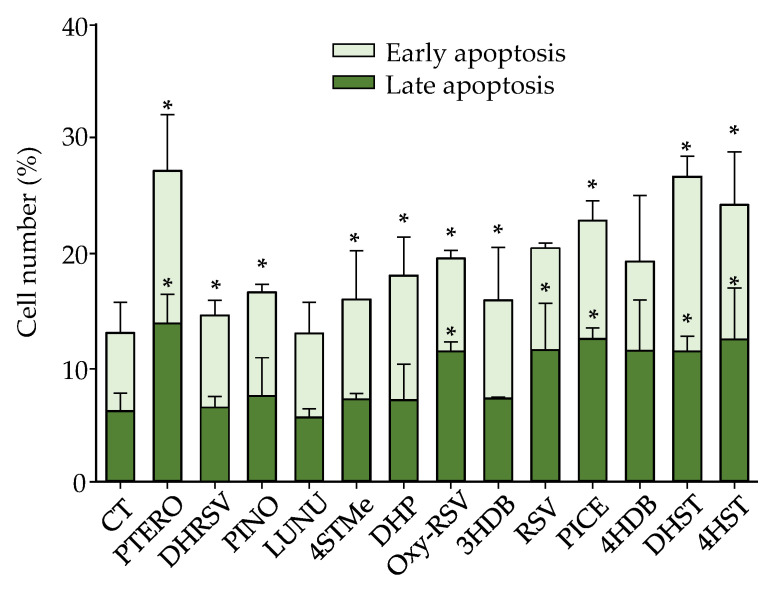
Apoptosis induction in Caco-2 cells after 72 h. IC_50_ concentrations (Table 1) were assayed for each compound. Data are shown as the mean ± SD of 3 independent experiments. * *p* < 0.05 vs. CT.

Regarding apoptosis induction, the treatments mostly induced a slight (8–12%) but statistically significant (*p* < 0.05) increase in the number of total (early plus late) apoptotic cells compared to control cells. However, no differences were observed between compounds (Figure 10). Finally, significant differences (*p* < 0.05) were found in the proportion of late-phase apoptotic cells vs. control cells for RSV, Oxy-RSV, PICE, PTERO, DHST, and 4HST, while for early-phase apoptotic cells, the increases were statistically significant (*p* < 0.05) for Oxy-RSV, PICE, PTERO, PINO, 3HDB, DHP, DHRSV, 4STMe, and DHST (Figure 10).

## 3. Discussion

When evaluating the in vitro anticancer potential of (poly)phenols and their derived metabolites, it is crucial to choose a plausible context compatible with the in vivo-occurring molecular forms and concentrations [19]. While phase-II-conjugated but not free (unconjugated) metabolites reach systemic tissues (breast, prostate, etc.), unconjugated compounds can only be extrapolated in terms of their physiological relevance in gastrointestinal cells, where interaction between some ingested phenolics and their gut microbial-derived metabolites can occur at relevant concentrations (high micromolar) [11,19]. Nevertheless, the specific cause–effect relationship between biological activity and the (poly)phenols ingested, including stilbenes and(or) their derived metabolites, is not fully understood [11]. In this regard, this gap is even more evident in the context of colorectal cancer [6]. While different studies have reported the in vivo activity of RSV against colorectal cancer, supported by their action mechanisms, the final driver of the effects has not been unequivocally identified [6,11]. This is especially relevant when human RSV metabolism differs depending on each individual’s gut microbiota, yielding the LUNU-producer and non-producer metabotypes, with a gradient of LUNU production within LUNU producers [15]. Therefore, the possible cancer chemopreventive effects of dietary RSV might differ in both human metabotypes, justifying the need for preclinical studies to explore this hypothesis.

**Figure 11:**
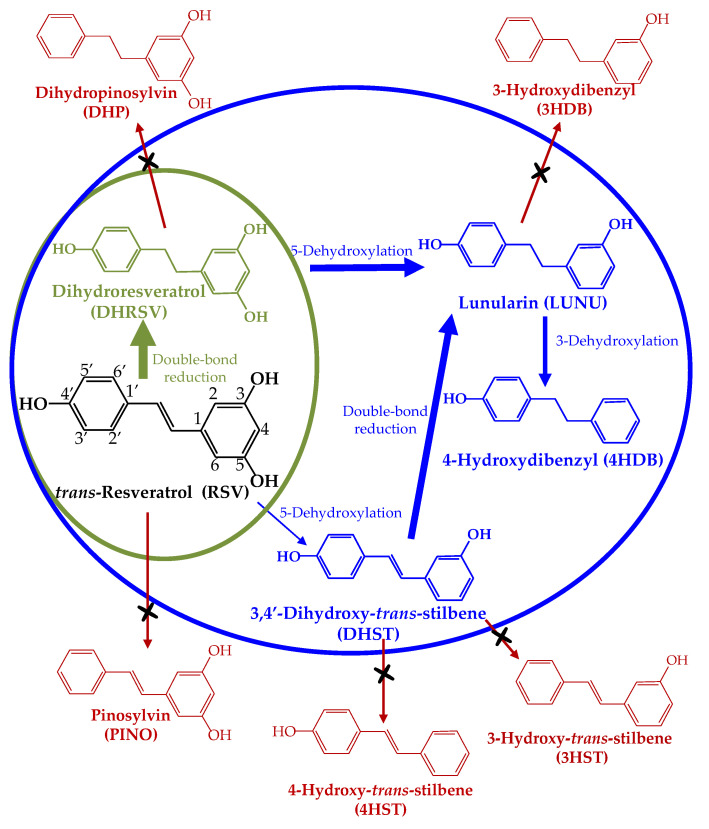
Metabolism of RSV by the human gut microbiota (adapted from [15]). Green circle, LUNU non-producer metabotype; blue circle, LUNU-producer metabotype; red compounds, metabolites not produced in humans [15].

Previous studies have compared the activity of stilbene analogs in colon cancer cells, but none have included stilbene and physiologically relevant dibenzyl metabolites derived from the gut microbiota. Therefore, in the present study, we have assayed RSV as the most representative dietary stilbene, and their gut microbial-derived metabolites DHRSV, DHST, LUNU, and 4HDB, representing the two recently reported gut microbiota metabotypes associated with RSV metabolism [15] (Figure 11). In addition, the dietary stilbenes PTERO, PICE, and Oxy-RSV [20,21], as well as other stilbene and dibenzyl analogs, were also assayed (Figure 1). Although some of these stilbenes and dibenzyls (i.e., 4HST, 3HDB, PINO, and DHP) are not produced by the human gut microbiota (Figure 11), they were assayed to explore the possible critical structural features related to the antiproliferative activity of stilbenes and dibenzyls against human colon cancer cells.

Comparing cancer and non-cancer cell lines is essential to rule out possible unwanted effects (cytotoxic) in non-tumor cells [19]. In general, the antiproliferative activity of phenolic compounds and their metabolites at equivalent doses is minimal or absent in various non-tumor cells, which do not present alterations in the cell cycle and also present lower detoxifying capacity than cancer cells [22,23]. However, in the present study, we observed antiproliferative effects exerted by DHRSV and LUNU in the CCD18-Co non-tumor colon cell line, compatible with a dietary context, which deserves further research to be confirmed.

Regarding the colon cancer cell lines, our results showed that reducing the stilbenes’ styrene double bond to yield the corresponding dibenzyls (e.g., catabolism from RSV to DHRSV or comparing 4HST vs. 4HDB) was critical to decreasing the antiproliferative activity in both Caco-2 and HT-29 cells. In addition, although the sequential dehydroxylation in stilbenes and dibenzyls decreased the anticancer activity, this property was maintained or even increased despite losing hydroxyl groups (–OH) if the double bond of the styrene core was preserved, probably because of the smaller size of the molecule (e.g., from RSV to DHST and from DHST to 4HST). In this regard, these extra –OH proved to hamper the entrance of RSV into melanoma cells and decreased the capacity to inhibit melanoma cell growth compared to 4HST [24]. Therefore, the 4′-hydroxystyryl moiety, although not essential, was associated with the highest antiproliferative effects, in agreement with previous reports in other cell lines [24,25,26]. Notably, the high glucuronidation capacity of HT-29 cells at the 4′-position could explain the higher resistance of these cells compared to Caco-2 cells, which showed lower metabolic activity and preferentially conjugated sulfate or glucuronyl groups at the 3-position. Therefore, our results confirm phase-II conjugations as critical modulators of the antiproliferative activity of stilbenes and dibenzyls as described for RSV and other phenolics and derived metabolites [27,28,29,30]. This fact may imply that, upon incubation time, some compounds may be more effectively metabolized and, thus, exert less effect as their reactive hydroxyl groups are blocked, suggesting that comparing activity with other studies should also consider the exposure time of the cells to the compounds. Therefore, a paradoxical situation could arise in which a molecule with a high possible antiproliferative potential (presence of several hydroxyl groups) could be efficiently metabolized by cancer cells similar to HT-29 and exert less effect than expected. In this regard, Nutakul et al. [31] compared the antiproliferative capacity of RSV and PTERO, obtaining IC_50_ values for HT-29 and Caco-2 cells that did not entirely coincide with the data obtained in our study. However, these authors only evaluated the activity after 24 h. In addition, they used the MTT method that could present some interferences with phenolic compounds [24,32]. In the present study, with a few exceptions, most compounds showed higher antiproliferative activity in Caco-2 than in HT-29 cells. In addition, a longer incubation time, from 48 to 72 h, slightly changed the activity order (results not shown), which seemed to be motivated by the capacity of HT-29 to metabolize the compounds and decrease their antiproliferative activity. These results could explain the heterogeneity of IC_50_ values at different incubation times described in the literature [31].

Overall, whether the catabolism of RSV is either a deactivation or an activation pathway deserves further research. For example, recent studies have described the catabolism of RSV by the gut microbiota to yield DHRSV and LUNU as a deactivation process since the resulting dibenzyls showed lower activity against insulin resistance and caloric restriction mimic properties [17]. In contrast, Li et al. [18] found that DHRSV and LUNU exhibited more potent anti-inflammatory and anticancer effects than RSV at the concentrations found in mouse tissues. However, these authors assayed 10-fold and 15-fold higher DHRSV and LUNU concentrations, respectively, than RSV in the cell models, explaining the different activities observed. Although the concentrations of the gut microbial-derived metabolites are usually higher than their precursors, relevant RSV concentrations have been described in human fecal samples and pig colon [15,33]. Overall, inherent to the miscellaneous health effects and associated mechanisms of phenolic-derived metabolites [34], we believe the higher or lower activity of these compounds should be analyzed independently for each specific process, i.e., inflammation, cancer, etc., and even depending on the particular assay model. In this regard, Lappano et al. [35] compared different stilbenes in estrogen-positive breast cancer cells and correlated the activity based on the ability of the compounds to bind to estrogen receptors. The activity and receptor binding affinity coincided, i.e., 3,4′-DHST > 4,4′-DHST > 4HST > RSV > PINO. Therefore, there is no universal antiproliferative activity sequence, and the cell type must always be considered. However, we must remind that this approach’s physiological relevance is questionable since neither RSV nor its microbial-derived metabolites reach human mammary tissues as free (unconjugated) compounds [36,37].

In the present study, the bi- and multivariate analyses showed no simple relationships between stilbene and dibenzyl features and IC_50_ values, suggesting interactions between different compound characteristics. For example, higher pKa1 values were generally associated with lower antiproliferative activity in Caco-2 cells, while no relationship was observed in HT-29 cells. This means that compounds with lower pKa1 values exerted higher antiproliferative activity in Caco-2 cells, indicating that the first hydroxyl group (4′-position) tends to dissociate more easily and increase its reactivity, as shown for RSV and PICE [38,39]. Notably, the metabolic preference of HT-29 cells for blocking precisely the 4′-position could explain the lack of relationship between pKa1 and IC_50_ values in HT-29 cells. Similarly, pKa2 will indicate how likely it is to dissociate the second –OH group, which will be (if there is more than one) located at the 3- or 5- positions [38,39]. This pKa2 also correlated with IC_50_ values in Caco-2 but not in HT-29 cells, probably due to the different metabolic capacities of both cell lines to block hydroxyl groups.

In the case of HT-29 cells, IC_50_ values were inversely and proportionally associated with LogP and LogS, respectively. These magnitudes are widely used in structure–activity studies [40] and predict the higher (LogS) or lower (LogP) solubility of a compound in an aqueous phase. In general, LogS could reflect a higher number of hydroxyl groups (the more hydroxylation, the higher Log S value), which, although it could confer more antiproliferative activity to the molecule [41], on the other hand, could also be better metabolized by the HT-29 cells [27]. In addition, the double bond in the styrene moiety confers hydrophobicity to the molecules (high LogP values), resulting in a paradoxical situation in which the smallest and most hydrophobic molecule (4HST), with only one hydroxyl group, exerted the highest antiproliferative activity against HT-29 cells.

Finally, Connolly’s solvent-excluded volume (i.e., the volume of space bounded by the molecular surface accessible to the solvent) and related features (Connolly’s molecular and accessible area) hindered the antiproliferative activity in both cell lines. Overall, the higher Connolly’s values refer to the larger size of the molecules. The inclusion of factors related to Connolly’s values reflects the contribution of the size of the molecule in models of absorption and the pass of the molecules through membranes, improving the prediction outcome in QSAR models [42].

When comparing the antiproliferative activity of the monohydroxylated stilbenes and dibenzyls assayed (Figure 1), 4HST was the most active compound in both cancer cell lines. Although the anticancer activity of this compound has been scarcely investigated, 4HST was described as the most active compound in a comparison of stilbenes against a melanoma cell line [24], revealing that the location of the hydroxyl group at the 4-position (4’ if there are more hydroxyls) is relevant to exert the activity, especially in stilbenes (4-hydroxystyryl moiety) [24,43]. In this regard, the double bond is critical for exerting a higher activity, i.e., 4HST vs. 4HDB, while the position of hydroxyl groups in dibenzyls (3-position vs. 4’-) did not show relevant differences in the activity (4HDB vs. 3HDB). Notably, the separation of the hydroxyl group from the aromatic ring (4STMe) hindered its ionization (higher pKa1) and reduced its activity, also confirming previous studies [24]. In general, the order from higher to lower activity in the monohydroxylated compounds was justified by their molecular features and the greater or lesser facility of the cells to block the hydroxyl groups yielding the corresponding phase-II conjugates [27]. Therefore, IC_50_ values reflected the activity of the compounds and their “resistance” to be metabolized by cells.

When the dihydroxylated compounds were compared (Figure 1), DHST vs. LUNU and DHST vs. DHP, as in the case of monohydroxylated molecules, the double bond was critical (more than the hydroxyl group position) in conferring higher activity (DHST). In addition, the correlation with LogS, LogP, and pKa1 values was also held.

In the case of trihydroxylated compounds, i.e., RSV and DHRSV, the double bond was the only structural determinant that could influence activity. As in the previous comparisons, the double bond in the styrene core of RSV conferred more activity against the two cancer cell lines, which was justified by its lower pKa1 (activity against Caco-2) and higher LogP (activity against HT-29).

Regarding the tetrahydroxystilbenes, the only difference was the position of a hydroxyl group at the 2’-position in Oxy-RSV vs. the 3’-position in PICE (Figure 1). In the two cancer cell lines, PICE exerted higher activity than Oxy-RSV, so the *o*-dihydroxyl configuration of PICE seemed responsible. In Caco-2, the higher PICE activity was also justified by its lower pKa1 value, and in HT-29, its higher hydrophobicity (higher LogP). A higher activity could be expected in these tetrahydroxystilbenes, especially in PICE, due to its *o*-diphenol group [41]. However, this catechol moiety may yield a higher instability in the cellular medium [41,44] and (or) the possibility of this group being inactivated by the enzyme catechol-*ortho*-methyl transferase (COMT). This enzyme is relevant in detoxifying xenobiotics, including PICE [44,45], which has been described in both cell lines, although more decisive in HT-29 by detoxifying phenolic compounds more effectively [46].

Finally, in the case of the dimethoxylated stilbene PTERO (Figure 1), it showed an intermediate-low antiproliferative activity in Caco-2, perhaps influenced by its relatively high pKa1 value. However, in HT-29 cells, PICE was the second most active, only behind 4HST, and justified by its high hydrophobicity (high LogP). PTERO has generally been described as a compound with high anticancer activity due to its high hydrophobicity, which translates into greater bioavailability and cell entry [20,31].

## 4. Materials and Methods

### 4.1. Reagents

Acetonitrile (CAN), dimethyl sulfoxide (DMSO), formic acid, and methanol (MeOH) were obtained from JT Baker (Deventer, The Netherlands). Ultrapure Millipore water was used throughout the study. The following chemicals were purchased from Sigma-Aldrich (St. Louis, MO, USA): Phosphate-buffered saline (PBS), trypan blue, MTT (3-(4,5-dimethylthiazol-2-yl)-2,5-diphenyltetrazolium bromide), *trans*-resveratrol (3,5,4′-trihydroxy-*trans*-stilbene, resveratrol, RSV, ≥99%), *trans*-pinosylvin (3,5-dihydroxy-*trans*-stilbene, PINO, 98%), and *trans*-4-stilbenemethanol (4STMe, ≈100%). Oxyresveratrol (3,5,2′,4′-tetrahydroxy-*trans*-stilbene, Oxy-RSV, >97%), piceatannol (3,5,3′,4′-tetrahydroxy-*trans*-stilbene, PICE, 99%), and pterostilbene (3,5-dimethoxy, 4′-hydroxy-*trans*-stilbene, PTERO, >97%) were obtained from Selleck Chemicals LCC (Houston, TX, USA). 4-Hydroxy-*trans*-stilbene (4HST, 98%) was obtained from ThermoFisher Sci. (Madrid, Spain). Dihydroresveratrol (DHRSV, >97%), 4-hydroxydibenzyl (4-(2-phenylethyl)phenol, 4HDB, >97%), 3-hydroxydibenzyl (3-(2-phenylethyl)phenol, 3HDB, >97%), 3,4′-dihydroxydibenzyl (3-(4-hydroxyphenethyl)phenol or lunularin, LUNU, >97%), dihydropinosylvin (or 3-hydroxy-(5-phenethyl)phenol, DHP, >97%), and 3,4′-dihydroxy-*trans*-stilbene (or 3-(4-hydroxystyryl)phenol, DHST, >97%) were synthesized as previously described [15,16]. RSV 4′-*O*-sulfate, RSV 3-*O*-glucuronide, DHRSV 3-*O*-glucuronide, and RSV 3-*O*-sulfate were obtained as described elsewhere [33].

### 4.2. Cell Lines, Cell Culture Conditions, and Treatments

The cell lines were obtained from the American Type Culture Collection (ATCC, Rockville, MD, USA) and cultured according to the ATCC. The human colon cancer cell lines Caco-2 (p53-null) and HT-29 (mutant p53), and non-tumorigenic colon cells CCD18-Co (wild-type p53) were grown as reported elsewhere [47]. All tested compounds were solubilized in DMSO (<0.5% in the culture medium) and filter-sterilized (0.2 μm) before addition to the culture media. The treatments consisted of a range of 8 concentrations from 0.78 to 100 μM to calculate the concentration that inhibited cell growth by 50% vs. control cells (0.5% DMSO), i.e., IC_50_ values, on the three cell lines at two incubation times (48 and 72 h). The effects of the compounds on Caco-2, HT-29, and CCD18-Co cell viability and proliferation were measured using the MTT reduction assay according to Giménez-Bastida et al. [47]. Data are presented as the mean ± standard deviation (SD) of at least three independent experiments (n = 2 wells for each compound (dose and time point) per experiment). Then, IC_50_ antiproliferative values were determined using a four-parameter logistic regression fit. The calculations were performed using the online tool Quest Graph^TM^ IC_50_ Calculator (AAT Bioquest, Inc., Sunnyvale, CA, USA (https://www.aatbio.com/tools/ic50-calculator, accessed on 26 April 2022).

### 4.3. Cell Cycle Distribution and Apoptosis Analysis

The effects of all the compounds at their corresponding IC_50_ values for 3 days of treatment on cell cycle distribution in Caco-2 cells were measured as described previously [48]. Data are shown as the mean ± SD of 3 independent experiments (2 wells per treatment) for each time point. In addition, the apoptosis induction exerted by all the compounds at their corresponding IC_50_ values for 3 days of treatment was examined using the Annexin V/PI detection kit (Molecular Probes, ThermoFisher Scientific, Madrid, Spain) as described previously [48]. Briefly, 20,000 Caco-2 cells per sample were analyzed by flow cytometry (Coulter, EPICS XL-MCL, Miami, FL, USA), and staurosporine 5 μM was used as a standard inducer of apoptosis. Finally, the percentage of alive (negative in both annexin V-FITC and PI), early apoptotic (positive in annexin V-FITC and negative in PI), late apoptotic (positive in both annexin V-FITC and PI), and necrotic cells (only positive in PI) was calculated. Data are shown as the mean ± SD of 3 independent experiments (2 wells per treatment) for each time point.

### 4.4. Metabolism of Stilbenes and Dibenzyls in Cancer Cell Lines

The culture media were collected and processed to determine the metabolism of some representative compounds assayed (RSV, DHRSV, PTERO, and 4HST) after incubation (0, 24, 48, and 72 h) in all cell lines at 50 μM of concentration as described elsewhere [23]. Briefly, cell culture supernatants were collected at the end of the experiment and analyzed to measure the presence and concentration of the tested compounds. First, ACN (200 μL) was added per 200 μL of culture media, vortexed, and centrifuged at 16,435× *g* for 10 min. The supernatant was then concentrated in a Speedvac^®^ concentrator (Savant SPD 121P), and then the residue was redissolved in 100 μL of MeOH and filtered (0.45 μm) before analysis by UPLC-QTOF-MS.

#### 4.4.1. UPLC-QTOF-MS Analyses

A previously validated method (linearity, precision, accuracy, limits of detection, and quantification) was used to analyze RSV and its derived metabolites [36]. Briefly, the analyses were performed on an Agilent 1290 Infinity UPLC system coupled to a 6550 Accurate-Mass quadrupole-time-of-flight (QTOF) mass spectrometer (Agilent Technologies, Waldbronn, Germany) using an electrospray interface (Jet Stream Technology). Chrysin was used as an internal control of the ionization signal. Spectra were acquired in the *m*/*z* range from 100 to 1100, in negative polarity mode (*m*/*z^−^*) and an acquisition rate of 1.5 spectra/s. Data were processed using the Mass Hunter Qualitative Analysis software (version B.06.00, Agilent). A targeted screening was used to identify possible phase-II metabolites (glucuronides, sulfates, and sulfoglucuronides) after RSV metabolism. In addition, MS/MS analysis provided additional information to achieve reliable compound identification. MS/MS product ion spectra were collected at *m*/*z* 50–800 range using a retention time window of 1 min, collision energy of 20 V, and acquisition rate of 4 spectra/s.

#### 4.4.2. Identification and Quantification of Metabolites

A direct comparison with the available standards was used to identify the different metabolites. In addition, their spectral properties, molecular mass, fragmentation pattern, and prediction score were used to identify some metabolites tentatively when no standards were available. The quantification of RSV and derived metabolites were determined by interpolation in the calibration curves obtained with their corresponding standards in the cell media. In addition, using extracted ion chromatograms (EICs) for area calculation and quantification reduced the possibility of misinterpreting overlapping peaks.

### 4.5. Analysis of the Molecular Structure of Stilbenes and Dibenzyls

The analysis was performed using the Chem3D Pro software 16.0.1.4. (Perkin Elmer Informatics Inc., Waltham, MA, USA). This program includes computational, descriptive, docking, and molecular prediction models. For pKa, LogS, and LogP, the software uses a computational model based on the Molecular Networks’ chemoinformatics platform MOSES (Molecular Networks GmbH, Erlangen, Germany) (Figure S4).

### 4.6. Statistics

The empirical distribution of data with the normality assumption was tested using the Shapiro–Wilk test. Significant differences among all IC_50_ values were calculated using one-way ANOVA (normal distribution) or ANOVA on Ranks (non-normal distribution) following the multiple comparisons Student–Newman–Keuls Method. Principal component plots in rotated space and heat maps were performed using SPSS Statistics v. 27.0.1.0 (IBM, Chicago, IL, USA) and MetaboAnalyst 5.0 (https://www.metaboanalyst.ca, accessed on 26 April 2022), respectively. Bivariate analyses were evaluated using Pearson or Spearman correlations to measure the strength and the direction of the relationship between compounds’ features and IC_50_ values. The cell cycle and apoptosis data comparison were evaluated using parametric statistics (Student’s *t*-test) or non-parametric statistics (Mann–Whitney U test) depending on the normal or non-normal data distribution. Graphics and figures were prepared using SigmaPlot 14.5 (Systat Software, San Jose, CA, USA) and MS Office Professional Plus 2016 (Microsoft, Redmond, WA, USA). Statistically significant differences were considered at * *p* < 0.05, ** *p* < 0.01, and *** *p* < 0.001.

## 5. Conclusions

For the first time, a structure–activity relationship study has compared the anticancer activity of a series of physiologically relevant stilbenes and dibenzyls derived from the gut microbiota and other structural analogs in colon cells. The antiproliferative effect was mediated by an arrest at the S phase, exerted by all compounds, or at the G_2_/M phase for PTERO and both phases in the case of 4HST and DHST. In addition, most compounds exerted a slight but significant increase in apoptosis.

In general, the antiproliferative activity decreased by reducing the styrene moiety’s double bond in stilbenes and the number of hydroxyl groups. This activity was influenced by several compound characteristics, especially the Connolly’s (molecular size), LogS, LogP (compounds solubility), and pKa1 values (dissociation of the hydroxyl group at the 4- or 4′-position), with a different impact depending on the cell line. The stilbene analog 4HST was the most active compound due to its small size, hydrophobicity, and preservation of the critical 4′-hydroxystyryl moiety despite having only one hydroxyl group in its molecule.

Low or no antiproliferative activity was observed in the non-tumor CCD18-Co colon cell line. Notably, the Caco-2 colon cancer cell line was more sensitive than the HT-29 colon cancer cell line for most of the compounds tested, which was mainly explained by the high efficiency of HT-29 for conjugating stilbenes and dibenzyls, especially in the most critically active related feature, the hydroxyl group at the 4- (or 4′-) position.

These results suggest that gut microbiota metabolism determines the antiproliferative effects of dietary stilbenes. Therefore, RSV consumption might exert different effects in individuals depending on their gut microbiota metabotypes associated with RSV metabolism, i.e., LUNU producers and non-producers, contributing to explaining the inter-individual variability of RSV health effects reported in the literature. In addition, our results highlight stilbenoid molecules, with small molecular size, and preserving the 4-hydroxystyryl group as critical requirements to maximize antiproliferative activity against colon cancer cells.

## Figures and Tables

**Table 1:** IC_50_ values ^a^ (μM) at 48 and 72 h in Caco-2 and HT-29 cells.

Compounds	Caco-2 (48 h)	HT-29 (48 h)	Caco-2 (72 h)	HT-29 (72 h)
4HST	26.9 ± 8.0	34.0 ± 8.1	11.4 ± 10.1	24.4 ± 11.3
RSV	50.4 ± 3.4	68.9 ± 22.6	18.8 ± 1.6	59.1 ± 9.9
DHST	57.1 ± 10.2	64.8 ± 15.6	22.7 ± 6.4	48.3 ± 17.8
PINO	55.3 ± 12.7	37.5 ± 14.3	26.4 ± 7.1	38.1 ± 8.0
DHRSV	55.4 ± 10.8	>100	33.8 ± 17.8	69.0 ± 9.3
PICE	55.4 ± 10.8	78.5 ± 5.7	42.0 ± 9.8	69.5 ± 4.6
3HDB	>100	>100	42.9 ± 4.1	81.7 ± 15.5
PTERO	67.6 ± 12.3	45.1 ± 13.7	43.0 ± 11.6	37.9 ± 5.4
LUNU	84.5 ± 11.7	>100	50.5 ± 23.1	53.2 ± 6.8
Oxy-RSV	74.7 ± 11.5	96.7 ± 2.4	55.4 ± 2.5	83.4 ± 5.8
4HDB	>100	>100	56.9 ± 8.0	96.7 ± 6.7
4STMe	88.3 ± 14.6	85.4 ± 16.5	59.9 ± 23.1	57.1 ± 18.9
DHP	54.0 ± 3.1	>100	73.9 ± 13.8	82.4 ± 12.1

^a^ Values are shown as mean ± SD (n = 3). Multiple comparisons to estimate significant differences among IC_50_ values are shown in Supplementary Materials (Table S1).

## Data Availability

The data presented in this study are available on request from the corresponding author.

## Supplementary Materials

The following supporting information can be downloaded at: https://www.mdpi.com/article/10.3390/ijms232315102/s1.
