# Supplementary material for: Main Determinants Affecting the Antiproliferative Activity of Stilbenes and Their Gut Microbiota Metabolites in Colon Cancer Cells: A Structure–Activity Relationship Study
Source: Int J Mol Sci. 2022 Dec 1;23(23):15102. doi: 10.3390/ijms232315102 (PMC9739882; doi:10.3390/ijms232315102)
Supplement: Supplementary file 1 [file ijms-23-15102-s001.zip › ijms-2028475-supplementary.pdf]

Supporting Information

Main Determinants Affecting the Antiproliferative Activity of Stilbenes and Their Gut Microbiota Metabolites in Colon Cancer Cells: A Structure–Activity Relationship Study

Antonio González-Sarrías [1], Juan Carlos Espín-Aguilar [1], Salvador Romero-Reyes [1], Julio Puigcerver [2], Mateo Alajarín [2], José Berná [2], María Victoria Selma [1] and Juan Carlos Espín [1,*]

1 Laboratory of Food and Health, Research Group on Quality, Safety, and Bioactivity of Plant Foods, Department of Food Science and Technology, CEBAS-CSIC, Campus de Espinardo, P.O. Box 164, 30100 Murcia, Spain

2 Department of Organic Chemistry, Faculty of Chemistry, University of Murcia, 30100 Murcia, Spain

* Correspondence: jcespin@cebas.csic.es

Table S1. Multiple comparisons of stilbene and dibenzyl IC_{50} values.

Caco-2 cells at 72 h (ANOVA on ranks followed by the Student-Newman-Keuls test).

Comparison	Diff of Ranks	q	*P*	Statistical significance
DHP *vs.* 4HST	173.000	5.708	0.002	Yes
DHP *vs.* RSV	159.000	5.679	0.002	Yes
DHP *vs.* DHST	146.000	5.683	0.002	Yes
DHP *vs.* PINO	132.000	5.646	0.002	Yes
DHP *vs.* DHRSV	114.000	5.410	0.004	Yes
DHP *vs.* PICE	85.000	4.531	0.030	Yes
DHP *vs.* 3HDB	85.000	5.167	0.005	Yes
DHP *vs.* PTERO	79.000	5.586	0.001	Yes
DHP *vs.* LUNU	62.000	5.240	0.002	Yes
DHP *vs.* 4STMe	33.000	3.466	0.068	No
DHP *vs.* 4HDB	32.000	4.438	0.005	Yes
DHP *vs.* Oxy-RSV	31.000	6.328	<0.001	Yes
Oxy-RSV *vs.* 4HST	142.000	5.071	0.016	Yes
Oxy-RSV *vs.* RSV	128.000	4.982	0.017	Yes
Oxy-RSV *vs.* DHST	115.000	4.919	0.017	Yes
Oxy-RSV *vs.* PINO	101.000	4.793	0.020	Yes
Oxy-RSV *vs.* DHRSV	83.000	4.424	0.037	Yes
Oxy-RSV *vs.* PICE	54.000	3.282	0.234	No
Oxy-RSV *vs.* 3HDB	54.000	3.818	0.075	No
Oxy-RSV *vs.* PTERO	48.000	4.057	0.034	Yes
Oxy-RSV *vs.* LUNU	31.000	3.256	0.098	No
Oxy-RSV *vs.* 4STMe	2.000	0.277	0.979	No
Oxy-RSV *vs.* 4HDB	1.000	0.204	0.885	No
4HDB *vs.* 4HST	141.000	5.488	0.004	Yes
4HDB *vs.* RSV	127.000	5.432	0.004	Yes
4HDB *vs.* DHST	114.000	5.410	0.004	Yes
4HDB *vs.* PINO	100.000	5.330	0.004	Yes
4HDB *vs.* DHRSV	82.000	4.984	0.008	Yes
4HDB *vs.* PICE	53.000	3.748	0.086	No
4HDB *vs.* 3HDB	53.000	4.479	0.013	Yes
4HDB *vs.* PTERO	47.000	4.936	0.003	Yes
4HDB *vs.* LUNU	30.000	4.160	0.009	Yes
4HDB *vs.* 4STMe	1.000	0.204	0.885	No
4STMe *vs.* 4HST	140.000	5.988	<0.001	Yes
4STMe *vs.* RSV	126.000	5.980	<0.001	Yes
4STMe *vs.* DHST	113.000	6.023	<0.001	Yes
4STMe *vs.* PINO	99.000	6.018	<0.001	Yes
4STMe *vs.* DHRSV	81.000	5.728	<0.001	Yes
4STMe *vs.* PICE	52.000	4.395	0.016	Yes
4STMe *vs.* 3HDB	52.000	5.461	<0.001	Yes
4STMe *vs.* PTERO	46.000	6.379	<0.001	Yes

4STMe *vs.* LUNU	29.000	5.920	<0.001	Yes
LUNU *vs.* 4HST	111.000	5.268	0.006	Yes
LUNU *vs.* RSV	97.000	5.170	0.006	Yes
LUNU *vs.* DHST	84.000	5.106	0.006	Yes
LUNU *vs.* PINO	70.000	4.950	0.006	Yes
LUNU *vs.* DHRSV	52.000	4.395	0.016	Yes
LUNU *vs.* PICE	23.000	2.415	0.319	No
LUNU *vs.* 3HDB	23.000	3.190	0.062	No
LUNU *vs.* PTERO	17.000	3.470	0.014	Yes
PTERO *vs.* 4HST	94.000	5.010	0.009	Yes
PTERO *vs.* RSV	80.000	4.863	0.011	Yes
PTERO *vs.* DHST	67.000	4.738	0.011	Yes
PTERO *vs.* PINO	53.000	4.479	0.013	Yes
PTERO *vs.* DHRSV	35.000	3.676	0.046	Yes
PTERO *vs.* PICE	6.000	0.832	0.826	No
PTERO *vs.* 3HDB	6.000	1.225	0.386	No
3HDB *vs.* 4HST	88.000	5.349	0.003	Yes
3HDB *vs.* RSV	74.000	5.233	0.003	Yes
3HDB *vs.* DHST	61.000	5.155	0.003	Yes
3HDB *vs.* PINO	47.000	4.936	0.003	Yes
3HDB *vs.* DHRSV	29.000	4.022	0.012	Yes
3HDB *vs.* PICE	0.000	0.000	1.000	No
PICE *vs.* 4HST	88.000	6.223	<0.001	Yes
PICE *vs.* RSV	74.000	6.254	<0.001	Yes
PICE *vs.* DHST	61.000	6.406	<0.001	Yes
PICE *vs.* PINO	47.000	6.518	<0.001	Yes
PICE *vs.* DHRSV	29.000	5.920	<0.001	Yes
DHRSV *vs.* 4HST	59.000	4.986	0.004	Yes
DHRSV *vs.* RSV	45.000	4.726	0.005	Yes
DHRSV *vs.* DHST	32.000	4.438	0.005	Yes
DHRSV *vs.* PINO	18.000	3.674	0.009	Yes
PINO *vs.* 4HST	41.000	4.306	0.012	Yes
PINO *vs.* RSV	27.000	3.744	0.022	Yes
PINO *vs.* DHST	14.000	2.858	0.043	Yes
DHST *vs.* 4HST	27.000	3.744	0.022	Yes
DHST *vs.* RSV	13.000	2.654	0.061	No
RSV *vs.* 4HST	14.000	2.858	0.043	Yes

HT-29 cells at 72 h (one-way ANOVA followed by the Student-Newman-Keuls test).

Comparison	Diff of Means	p	q	*P*	Statistical significance
4HDB *vs.* 4HST	72.352	13	12.970	<0.001	Yes
4HDB *vs.* PTERO	58.836	12	10.547	<0.001	Yes
4HDB *vs.* PINO	58.578	11	10.501	<0.001	Yes
4HDB *vs.* DHST	48.380	10	8.673	<0.001	Yes
4HDB *vs.* LUNU	43.473	9	7.793	<0.001	Yes
4HDB *vs.* 4STMe	39.593	8	7.098	<0.001	Yes
4HDB *vs.* RSV	37.573	7	6.735	<0.001	Yes

4HDB *vs.* DHRSV	27.736	6	4.972	0.013	Yes
4HDB *vs.* PICE	27.231	5	4.882	0.011	Yes
4HDB *vs.* 3HDB	14.997	4	2.688	0.244	No
4HDB *vs.* DHP	14.314	3	2.566	0.178	No
4HDB *vs.* Oxy-RSV	13.331	2	2.390	0.099	No
Oxy-RSV *vs.* 4HST	59.022	12	10.581	<0.001	Yes
Oxy-RSV *vs.* PTERO	45.506	11	8.158	<0.001	Yes
Oxy-RSV *vs.* PINO	45.247	10	8.111	<0.001	Yes
Oxy-RSV *vs.* DHST	35.049	9	6.283	0.002	Yes
Oxy-RSV *vs.* LUNU	30.143	8	5.404	0.010	Yes
Oxy-RSV *vs.* 4STMe	26.262	7	4.708	0.029	Yes
Oxy-RSV *vs.* RSV	24.242	6	4.346	0.042	Yes
Oxy-RSV *vs.* DHRSV	14.405	5	2.582	0.374	No
Oxy-RSV *vs.* PICE	13.901	4	2.492	0.307	No
Oxy-RSV *vs.* 3HDB	1.667	3	0.299	0.976	No
Oxy-RSV *vs.* DHP	0.984	2	0.176	0.902	No
DHP *vs.* 4HST	58.038	11	10.404	<0.001	Yes
DHP *vs.* PTERO	44.522	10	7.981	<0.001	Yes
DHP *vs.* PINO	44.264	9	7.935	<0.001	Yes
DHP *vs.* DHST	34.066	8	6.107	0.003	Yes
DHP *vs.* LUNU	29.159	7	5.227	0.011	Yes
DHP *vs.* 4STMe	25.279	6	4.532	0.030	Yes
DHP *vs.* RSV	23.259	5	4.169	0.041	Yes
DHP *vs.* DHRSV	13.421	4	2.406	0.337	No
DHP *vs.* PICE	12.917	3	2.316	0.242	No
DHP *vs.* 3HDB	0.683	2	0.122	0.932	No
3HDB *vs.* 4HST	57.355	10	10.282	<0.001	Yes
3HDB *vs.* PTERO	43.839	9	7.859	<0.001	Yes
3HDB *vs.* PINO	43.581	8	7.812	<0.001	Yes
3HDB *vs.* DHST	33.383	7	5.984	0.003	Yes
3HDB *vs.* LUNU	28.476	6	5.105	0.011	Yes
3HDB *vs.* 4STMe	24.596	5	4.409	0.027	Yes
3HDB *vs.* RSV	22.575	4	4.047	0.033	Yes
3HDB *vs.* DHRSV	12.738	3	2.284	0.252	No
3HDB *vs.* PICE	12.234	2	2.193	0.129	No
PICE *vs.* 4HST	45.121	9	8.089	<0.001	Yes
PICE *vs.* PTERO	31.605	8	5.666	0.006	Yes
PICE *vs.* PINO	31.347	7	5.619	0.005	Yes
PICE *vs.* DHST	21.149	6	3.791	0.102	No
PICE *vs.* LUNU	16.242	5	2.912	0.258	No
PICE *vs.* 4STMe	12.362	4	2.216	0.409	No
PICE *vs.* RSV	10.341	3	1.854	0.398	No
PICE *vs.* DHRSV	0.504	2	0.0904	0.949	No
DHRSV *vs.* 4HST	44.617	8	7.998	<0.001	Yes
DHRSV *vs.* PTERO	31.101	7	5.575	0.006	Yes
DHRSV *vs.* PINO	30.842	6	5.529	0.005	Yes
DHRSV *vs.* DHST	20.644	5	3.701	0.087	No
DHRSV *vs.* LUNU	15.738	4	2.821	0.207	No

DHRSV *vs.* 4STMe	11.857	3	2.126	0.301	No
DHRSV *vs.* RSV	9.837	2	1.763	0.220	No
RSV *vs.* 4HST	34.779	7	6.235	0.002	Yes
RSV *vs.* PTERO	21.264	6	3.812	0.099	No
RSV *vs.* PINO	21.005	5	3.766	0.079	No
RSV *vs.* DHST	10.807	4	1.937	0.525	No
RSV *vs.* LUNU	5.901	3	1.058	0.737	No
RSV *vs.* 4STMe	2.020	2	0.362	0.799	No
4STMe *vs.* 4HST	32.759	6	5.873	0.002	Yes
4STMe *vs.* PTERO	19.243	5	3.450	0.126	No
4STMe *vs.* PINO	18.985	4	3.403	0.093	No
4STMe *vs.* DHST	8.787	3	1.575	0.512	No
4STMe *vs.* LUNU	3.880	2	0.696	0.626	No
LUNU *vs.* 4HST	28.879	5	5.177	0.006	Yes
LUNU *vs.* PTERO	15.363	4	2.754	0.226	No
LUNU *vs.* PINO	15.105	3	2.708	0.148	No
LUNU *vs.* DHST	4.907	2	0.880	0.538	No
DHST *vs.* 4HST	23.972	4	4.297	0.021	Yes
DHST *vs.* PTERO	10.456	3	1.874	0.390	No
DHST *vs.* PINO	10.198	2	1.828	0.204	No
PINO *vs.* 4HST	13.774	3	2.469	0.201	No
PINO *vs.* PTERO	0.258	2	0.0463	0.974	No
PTERO *vs.* 4HST	13.516	2	2.423	0.095	No

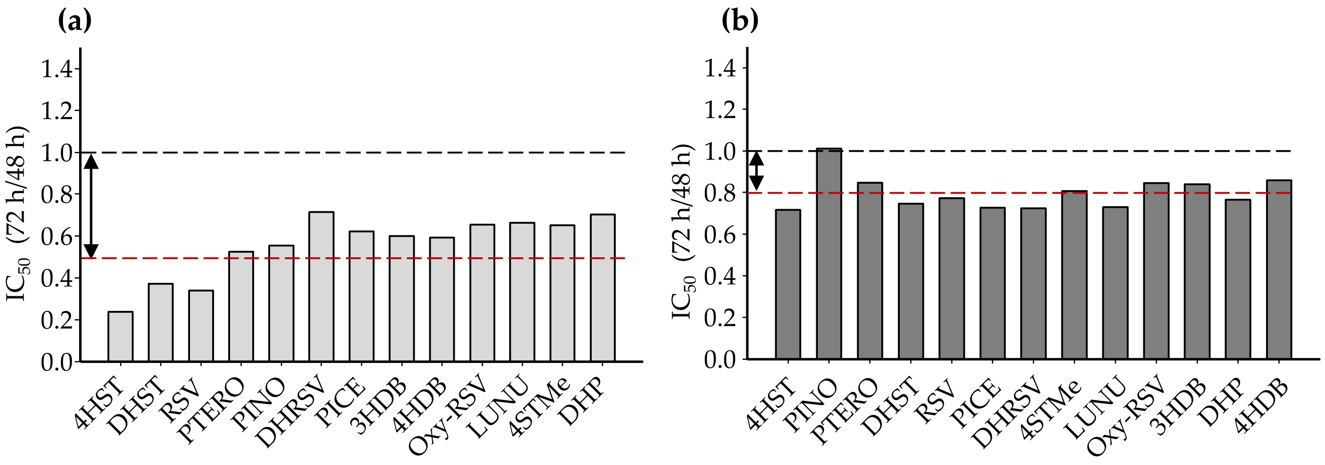

Figure S1. Mean decrease of IC_{50} values from 48 to 72 h of incubation in Caco-2 (**a**) and HT-29 (**b**) cells. Black dashed lines: no change; Red dashed lines: mean IC_{50} reduction. IC_{50} values decreased 2-fold in Caco-2 (**a**) and 1.25-fold (**b**) in HT-29 cells.

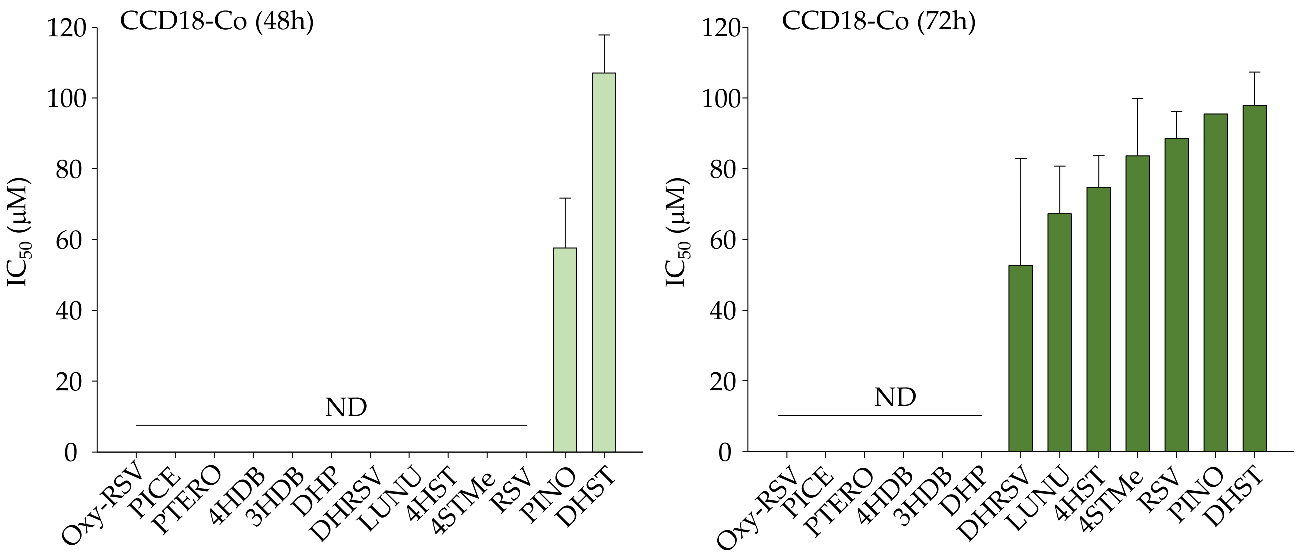

Figure S2. Antiproliferative IC_{50} values of stilbenes and dibenzyls in the non-tumorigenic CCD18-Co cell line at 48 and 72 h. ND: not detected. Values are shown as mean ± SD (n=3).

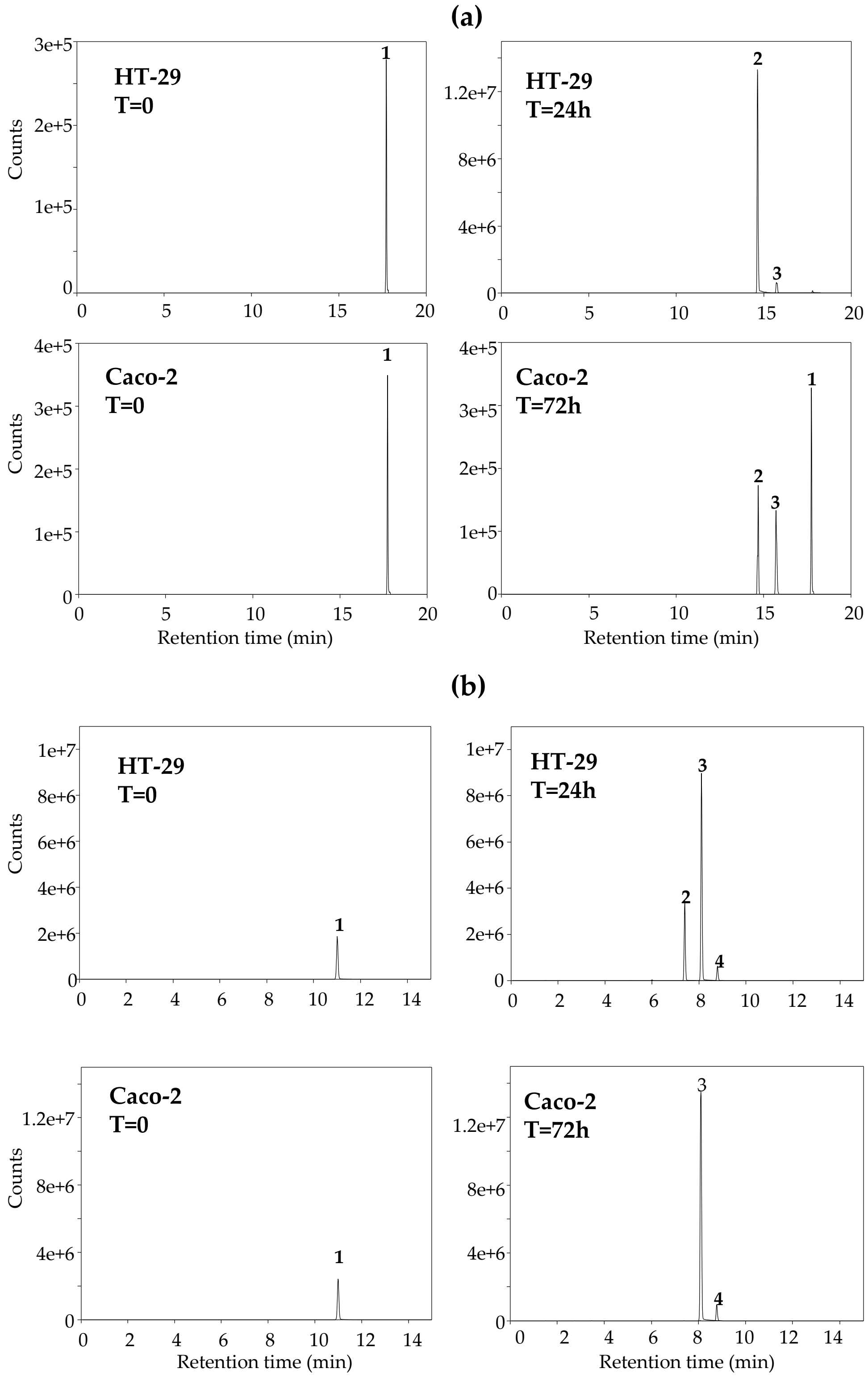

Figure S3. Extracted ion chromatograms showing the phase-II metabolism of pterostilbene (PTERO) (**a**) and dihydroresveratrol (DHRSV) (**b**) by HT-29 (0 and 24 h) and Caco-2 cells (0 and 72 h). (**a**): PTERO (**1**, m/z^- 255.1027), PTERO glucuronide (**2**, m/z^- 431.1348), and PTERO sulfate (**3**, m/z^- 335.0595); (**b**): DHRSV (**1**, m/z^- 229.087), DHRSV 4′-*O*-glucuronide (**2**, m/z^- 405.1191), DHRSV 3-*O*-glucuronide (**3**, m/z^- 405.1191), and DHRSV 3-*O*-sulfate (**4**, m/z^- 309.0438).

Figure S4. Molecular characteristics of stilbenes and dibenzyls (Chem3D Pro).

4-Hydroxydibenzyl (4HDB)

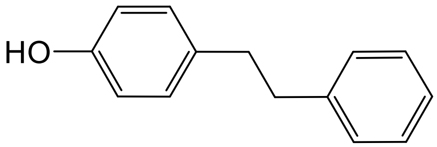

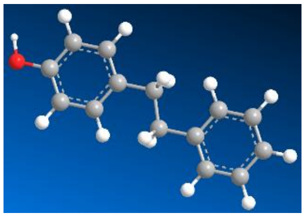

ChemPropStd: Formal Charge = 0
ChemPropStd: Connolly Accessible Area = 414.834 Å^2
ChemPropStd: Connolly Molecular Area = 210.781 Å^2
ChemPropStd: Connolly Solvent Excluded Volume = 173.699 Å^3
ChemPropStd: Exact Mass = 198.1044650715 g/Mol
ChemPropStd: Mass = 198.26500000145
ChemPropStd: Mol Weight = 198.26500000145
ChemPropStd: Number of HBond Acceptors = 1
ChemPropStd: Number of HBond Donors = 1
ChemPropStd: Ovality = 1.4000679822941
ChemPropStd: Principal Moment = 200.416 2300.665 2494.471
ChemPropStd: Elemental Analysis = C, 84.81; H, 7.12; O, 8.07
ChemPropStd: m/z = 198.10 (100.0%), 199.11 (15.1%), 200.11 (1.1%)
ChemPropStd: Mol Formula = C14H14O
ChemPropStd: Mol Formula HTML = C14H14O
CLogP Driver: Mol Refractivity = 6.28049993515015
CLogP Driver: Partition Coefficient = 3.92100024223328
Molecular Networks: LogP = 3.96856 Log Units
Molecular Networks: LogS = -3.67827 Log Units
Molecular Networks: pKa = pKa1: 9.69656 Log Units
Molecular Topology: Balaban Index = 66628
Molecular Topology: Cluster Count = 15
Molecular Topology: Molecular Topological Index = 3332
Molecular Topology: Num Rotatable Bonds = 3 Bond(s)
Molecular Topology: Polar Surface Area = 20.23 Å^2
Molecular Topology: Radius = 5 Atom(s)
Molecular Topology: Shape Attribute = 13.0666666666667
Molecular Topology: Shape Coefficient = 1
Molecular Topology: Sum Of Degrees = 32
Molecular Topology: Sum Of Valence Degrees = 48
Molecular Topology: Topological Diameter = 10 Bond(s)
Molecular Topology: Total Connectivity = 0.00425258635899857
Molecular Topology: Total Valence Connectivity = 0.000199227538385155
Molecular Topology: Wiener Index = 420

3-Hydroxydibenzyl (3HDB)

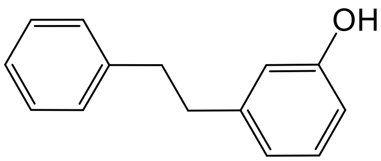

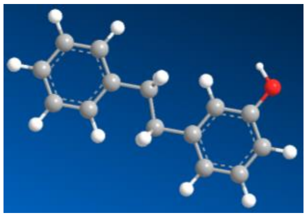

ChemPropStd: Formal Charge = 0
ChemPropStd: Connolly Accessible Area = 414.844 Å^2
ChemPropStd: Connolly Molecular Area = 210.786 Å^2
ChemPropStd: Connolly Solvent Excluded Volume = 173.702 Å^3
ChemPropStd: Exact Mass = 198.1044650715 g/Mol
ChemPropStd: Mass = 198.26500000145
ChemPropStd: Mol Weight = 198.26500000145
ChemPropStd: Number of HBond Acceptors = 1
ChemPropStd: Number of HBond Donors = 1
ChemPropStd: Ovality = 1.40008507295851
ChemPropStd: Principal Moment = 284.504 2040.581 2318.474
ChemPropStd: Elemental Analysis = C, 84.81; H, 7.12; O, 8.07
ChemPropStd: m/z = 198.10 (100.0%), 199.11 (15.1%), 200.11 (1.1%)
ChemPropStd: Mol Formula = C14H14O
ChemPropStd: Mol Formula HTML = C14H14O
CLogP Driver: Mol Refractivity = 6.28049993515015
CLogP Driver: Partition Coefficient = 3.92100024223328
Molecular Networks: LogP = 3.96856 Log Units
Molecular Networks: LogS = -3.68537 Log Units
Molecular Networks: pKa = pKa1: 9.69848 Log Units
Molecular Topology: Balaban Index = 65403
Molecular Topology: Cluster Count = 15
Molecular Topology: Molecular Topological Index = 3284
Molecular Topology: Num Rotatable Bonds = 3 Bond(s)
Molecular Topology: Polar Surface Area = 20.23 Å^2
Molecular Topology: Radius = 5 Atom(s)
Molecular Topology: Shape Attribute = 13.0666666666667
Molecular Topology: Shape Coefficient = 0
Molecular Topology: Sum Of Degrees = 32
Molecular Topology: Sum Of Valence Degrees = 48
Molecular Topology: Topological Diameter = 9 Bond(s)
Molecular Topology: Total Connectivity = 0.00425258635899857
Molecular Topology: Total Valence Connectivity = 0.000199227538385155
Molecular Topology: Wiener Index = 412

4-Hydroxy-*trans*-stilbene (4HST)

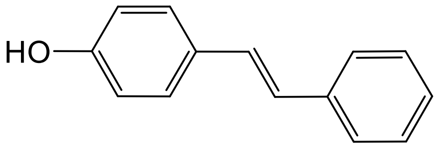

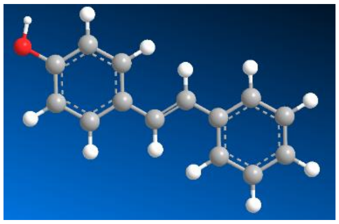

ChemPropStd: Formal Charge = 0
ChemPropStd: Connolly Accessible Area = 400.958 Å^2
ChemPropStd: Connolly Molecular Area = 201.86 Å^2
ChemPropStd: Connolly Solvent Excluded Volume = 160.745 Å^3
ChemPropStd: Exact Mass = 196.0888150073 g/Mol
ChemPropStd: Mass = 196.24900000135
ChemPropStd: Mol Weight = 196.24900000135
ChemPropStd: Number of HBond Acceptors = 1
ChemPropStd: Number of HBond Donors = 1
ChemPropStd: Ovality = 1.41191264934254
ChemPropStd: Principal Moment = 192.525 2174.135 2366.661
ChemPropStd: Elemental Analysis = C, 85.68; H, 6.16; O, 8.15
ChemPropStd: m/z = 196.09 (100.0%), 197.09 (15.1%), 198.10 (1.1%)
ChemPropStd: Mol Formula = C14H12O
ChemPropStd: Mol Formula HTML = C14H12O
CLogP Driver: Mol Refractivity = 6.40709972381592
CLogP Driver: Partition Coefficient = 4.16699981689453
Molecular Networks: LogP = 4.24354 Log Units
Molecular Networks: LogS = -3.98452 Log Units
Molecular Networks: pKa = pKa1: 9.54252 Log Units
Molecular Topology: Balaban Index = 66628
Molecular Topology: Cluster Count = 15
Molecular Topology: Molecular Topological Index = 3332
Molecular Topology: Num Rotatable Bonds = 2 Bond(s)
Molecular Topology: Polar Surface Area = 20.23 Å^2
Molecular Topology: Radius = 5 Atom(s)
Molecular Topology: Shape Attribute = 13.0666666666667
Molecular Topology: Shape Coefficient = 1
Molecular Topology: Sum Of Degrees = 32
Molecular Topology: Sum Of Valence Degrees = 50
Molecular Topology: Topological Diameter = 10 Bond(s)
Molecular Topology: Total Connectivity = 0.00425258635899857
Molecular Topology: Total Valence Connectivity = 0.000132818358923437
Molecular Topology: Wiener Index = 420 ---

***trans*-4-Stilbenemethanol (4STMe)**

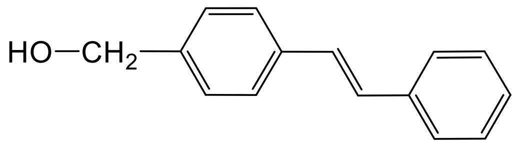

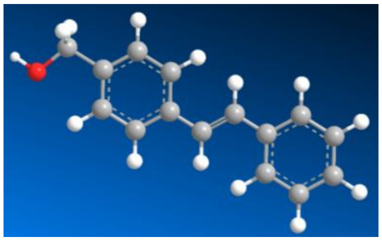

ChemPropStd: Formal Charge = 0
ChemPropStd: Connolly Accessible Area = 432.521 Å^2
ChemPropStd: Connolly Molecular Area = 220.701 Å^2
ChemPropStd: Connolly Solvent Excluded Volume = 177.58 Å^3
ChemPropStd: Exact Mass = 210.1044650715 g/Mol
ChemPropStd: Mass = 210.2760000015
ChemPropStd: Mol Weight = 210.2760000015
ChemPropStd: Number of HBond Acceptors = 1
ChemPropStd: Number of HBond Donors = 1
ChemPropStd: Ovality = 1.44452193855068
ChemPropStd: Principal Moment = 202.135 2733.524 2932.353
ChemPropStd: Elemental Analysis = C, 85.68; H, 6.71; O, 7.61
ChemPropStd: m/z = 210.10 (100.0%), 211.11 (16.2%), 212.11 (1.2%)
ChemPropStd: Mol Formula = C15H14O
ChemPropStd: Mol Formula HTML = C15H14O
CLogP Driver: Mol Refractivity = 6.87089967727661
CLogP Driver: Partition Coefficient = 3.7960000038147
Molecular Networks: LogP = 3.84042 Log Units
Molecular Networks: LogS = -3.72757 Log Units
Molecular Networks: pKa = pKa1: 14.3205 Log Units
Molecular Topology: Balaban Index = 91948
Molecular Topology: Cluster Count = 16
Molecular Topology: Molecular Topological Index = 4046
Molecular Topology: Num Rotatable Bonds = 3 Bond(s)
Molecular Topology: Polar Surface Area = 20.23 Å^2
Molecular Topology: Radius = 6 Atom(s)
Molecular Topology: Shape Attribute = 14.0625
Molecular Topology: Shape Coefficient = 0
Molecular Topology: Sum Of Degrees = 34
Molecular Topology: Sum Of Valence Degrees = 52
Molecular Topology: Topological Diameter = 11 Bond(s)
Molecular Topology: Total Connectivity = 0.0030070326520293
Molecular Topology: Total Valence Connectivity = 9.3916762260831 E-05
Molecular Topology: Wiener Index = 512

Lunularin (LUNU)

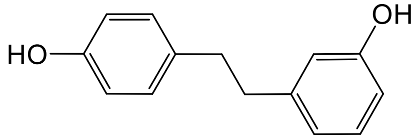

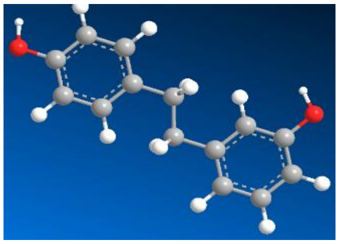

ChemPropStd: Formal Charge = 0
ChemPropStd: Connolly Accessible Area = 426.29 Å^2
ChemPropStd: Connolly Molecular Area = 218.184 Å^2
ChemPropStd: Connolly Solvent Excluded Volume = 180.363 Å^3
ChemPropStd: Exact Mass = 214.0993796936 g/Mol
ChemPropStd: Mass = 214.2640000015
ChemPropStd: Mol Weight = 214.2640000015
ChemPropStd: Number of HBond Acceptors = 2
ChemPropStd: Number of HBond Donors = 2
ChemPropStd: Ovality = 1.41331990239692
ChemPropStd: Principal Moment = 285.912 2594.569 2873.871
ChemPropStd: Elemental Analysis = C, 78.48; H, 6.59; O, 14.93
ChemPropStd: m/z = 214.10 (100.0%), 215.10 (15.1%), 216.11 (1.1%)
ChemPropStd: Mol Formula = C14H14O2
ChemPropStd: Mol Formula HTML = C14H14O2
CLogP Driver: Mol Refractivity = 6.43359994888306
CLogP Driver: Partition Coefficient = 3.25399994850159
Molecular Networks: LogP = 3.14367 Log Units
Molecular Networks: LogS = -3.33896 Log Units
Molecular Networks: pKa = pKa1: 9.78843, pKa2: 9.69251 Log Units
Molecular Topology: Balaban Index = 89726
Molecular Topology: Cluster Count = 16
Molecular Topology: Molecular Topological Index = 3792
Molecular Topology: Num Rotatable Bonds = 3 Bond(s)
Molecular Topology: Polar Surface Area = 40.46 Å^2
Molecular Topology: Radius = 5 Atom(s)
Molecular Topology: Shape Attribute = 14.0625
Molecular Topology: Shape Coefficient = 1
Molecular Topology: Sum Of Degrees = 34
Molecular Topology: Sum Of Valence Degrees = 54
Molecular Topology: Topological Diameter = 10 Bond(s)
Molecular Topology: Total Connectivity = 0.00347222222222222
Molecular Topology: Total Valence Connectivity = 7.71604938271605E-05
Molecular Topology: Wiener Index = 499

3,4′-Dihydroxy-*trans*-stilbene (DHST)

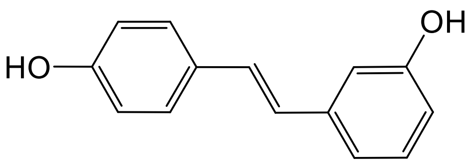

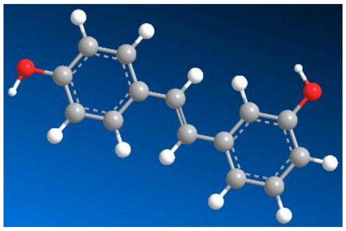

ChemPropStd: Formal Charge = 0
ChemPropStd: Connolly Accessible Area = 412.415 Å^2
ChemPropStd: Connolly Molecular Area = 209.261 Å^2
ChemPropStd: Connolly Solvent Excluded Volume = 167.405 Å^3
ChemPropStd: Exact Mass = 212.0837296294 g/Mol
ChemPropStd: Mass = 212.2480000014
ChemPropStd: Mol Weight = 212.2480000014
ChemPropStd: Number of HBond Acceptors = 2
ChemPropStd: Number of HBond Donors = 2
ChemPropStd: Ovality = 1.42459655042544
ChemPropStd: Principal Moment = 274.919 2467.460 2742.379
ChemPropStd: Elemental Analysis = C, 79.23; H, 5.70; O, 15.08
ChemPropStd: m/z = 212.08 (100.0%), 213.09 (15.1%), 214.09 (1.1%)
ChemPropStd: Mol Formula = C14H12O2
ChemPropStd: Mol Formula HTML = C14H12O2
CLogP Driver: Mol Refractivity = 6.56019973754883
CLogP Driver: Partition Coefficient = 3.5
Molecular Networks: LogP = 3.41865 Log Units
Molecular Networks: LogS = -3.64489 Log Units
Molecular Networks: pKa = pKa1: 9.29009, pKa2: 9.51862 Log Units
Molecular Topology: Balaban Index = 89726
Molecular Topology: Cluster Count = 16
Molecular Topology: Molecular Topological Index = 3792
Molecular Topology: Num Rotatable Bonds = 2 Bond(s)
Molecular Topology: Polar Surface Area = 40.46 Å^2
Molecular Topology: Radius = 5 Atom(s)
Molecular Topology: Shape Attribute = 14.0625
Molecular Topology: Shape Coefficient = 1
Molecular Topology: Sum Of Degrees = 34
Molecular Topology: Sum Of Valence Degrees = 56
Molecular Topology: Topological Diameter = 10 Bond(s)
Molecular Topology: Total Connectivity = 0.00347222222222222
Molecular Topology: Total Valence Connectivity = 5.1440329218107 E-05
Molecular Topology: Wiener Index = 499

Dihydropinosylvin (DHP)

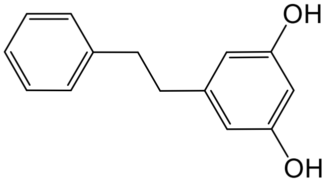

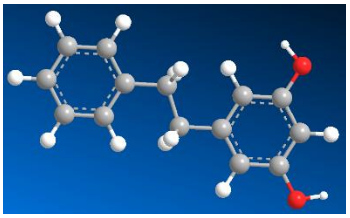

ChemPropStd: Formal Charge = 0
ChemPropStd: Connolly Accessible Area = 426.286 Å^2
ChemPropStd: Connolly Molecular Area = 218.182 Å^2
ChemPropStd: Connolly Solvent Excluded Volume = 180.356 Å^3
ChemPropStd: Exact Mass = 214.0993796936 g/Mol
ChemPropStd: Mass = 214.2640000015
ChemPropStd: Mol Weight = 214.2640000015
ChemPropStd: Number of HBond Acceptors = 2
ChemPropStd: Number of HBond Donors = 2
ChemPropStd: Ovality = 1.41334351582341
ChemPropStd: Principal Moment = 363.882 2359.513 2716.785
ChemPropStd: Elemental Analysis = C, 78.48; H, 6.59; O, 14.93
ChemPropStd: m/z = 214.10 (100.0%), 215.10 (15.1%), 216.11 (1.1%)
ChemPropStd: Mol Formula = C14H14O2
ChemPropStd: Mol Formula HTML = C14H14O2
CLogP Driver: Mol Refractivity = 6.43359994888306
CLogP Driver: Partition Coefficient = 3.25399994850159
Molecular Networks: LogP = 3.55958 Log Units
Molecular Networks: LogS = -3.63411 Log Units
Molecular Networks: pKa = pKa1: 9.54456, pKa2: 9.54456 Log Units
Molecular Topology: Balaban Index = 87246
Molecular Topology: Cluster Count = 16
Molecular Topology: Molecular Topological Index = 3720
Molecular Topology: Num Rotatable Bonds = 3 Bond(s)
Molecular Topology: Polar Surface Area = 40.46 Å^2
Molecular Topology: Radius = 5 Atom(s)
Molecular Topology: Shape Attribute = 14.0625
Molecular Topology: Shape Coefficient = 0
Molecular Topology: Sum Of Degrees = 34
Molecular Topology: Sum Of Valence Degrees = 54
Molecular Topology: Topological Diameter = 9 Bond(s)
Molecular Topology: Total Connectivity = 0.00347222222222222
Molecular Topology: Total Valence Connectivity = 7.71604938271605 E-05
Molecular Topology: Wiener Index = 485

Pinosylvin (PINO)

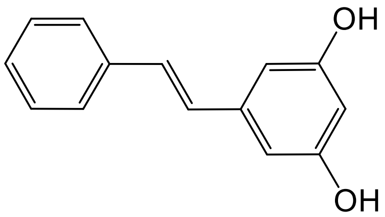

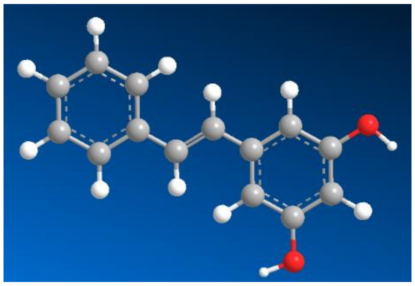

ChemPropStd: Formal Charge = 0
ChemPropStd: Connolly Accessible Area = 412.427 Å^2
ChemPropStd: Connolly Molecular Area = 209.267 Å^2
ChemPropStd: Connolly Solvent Excluded Volume = 167.411 Å^3
ChemPropStd: Exact Mass = 212.0837296294 g/Mol
ChemPropStd: Mass = 212.2480000014
ChemPropStd: Mol Weight = 212.2480000014
ChemPropStd: Number of HBond Acceptors = 2
ChemPropStd: Number of HBond Donors = 2
ChemPropStd: Ovality = 1.42460335744336
ChemPropStd: Principal Moment = 357.720 2225.664 2583.384
ChemPropStd: Elemental Analysis = C, 79.23; H, 5.70; O, 15.08
ChemPropStd: m/z = 212.08 (100.0%), 213.09 (15.1%), 214.09 (1.1%)
ChemPropStd: Mol Formula = C14H12O2
ChemPropStd: Mol Formula HTML = C14H12O2
CLogP Driver: Mol Refractivity = 6.56019973754883
CLogP Driver: Partition Coefficient = 3.5
Molecular Networks: LogP = 3.83456 Log Units
Molecular Networks: LogS = -3.93028 Log Units
Molecular Networks: pKa = pKa1: 9.05838, pKa2: 9.05838 Log Units
Molecular Topology: Balaban Index = 87246
Molecular Topology: Cluster Count = 16
Molecular Topology: Molecular Topological Index = 3720
Molecular Topology: Num Rotatable Bonds = 2 Bond(s)
Molecular Topology: Polar Surface Area = 40.46 Å^2
Molecular Topology: Radius = 5 Atom(s)
Molecular Topology: Shape Attribute = 14.0625
Molecular Topology: Shape Coefficient = 0
Molecular Topology: Sum Of Degrees = 34
Molecular Topology: Sum Of Valence Degrees = 56
Molecular Topology: Topological Diameter = 9 Bond(s)
Molecular Topology: Total Connectivity = 0.00347222222222222
Molecular Topology: Total Valence Connectivity = 5.1440329218107 E-05
Molecular Topology: Wiener Index = 485

***trans*-Resveratrol (RSV)**

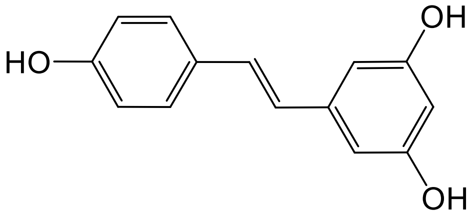

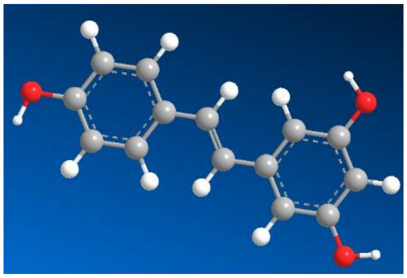

ChemPropStd: Formal Charge = 0
ChemPropStd: Connolly Accessible Area = 423.868 Å^2
ChemPropStd: Connolly Molecular Area = 216.66 Å^2
ChemPropStd: Connolly Solvent Excluded Volume = 174.062 Å^3
ChemPropStd: Exact Mass = 228.0786442515 g/Mol
ChemPropStd: Mass = 228.24700000145
ChemPropStd: Mol Weight = 228.24700000145
ChemPropStd: Number of HBond Acceptors = 3
ChemPropStd: Number of HBond Donors = 3
ChemPropStd: Ovality = 1.43711647490366
ChemPropStd: Principal Moment = 362.982 2815.846 3178.829
ChemPropStd: Elemental Analysis = C, 73.67; H, 5.30; O, 21.03
ChemPropStd: m/z = 228.08 (100.0%), 229.08 (15.1%), 230.09 (1.1%)
ChemPropStd: Mol Formula = C14H12O3
ChemPropStd: Mol Formula HTML = C14H12O3
CLogP Driver: Mol Refractivity = 6.71329975128174
CLogP Driver: Partition Coefficient = 2.83299994468689
Molecular Networks: LogP = 2.59376 Log Units
Molecular Networks: LogS = -3.29862 Log Units
Molecular Networks: pKa = pKa1: 9.1338, pKa2: 9.50554, pKa3:9.50554 Log Units
Molecular Topology: Balaban Index = 117735
Molecular Topology: Cluster Count = 17
Molecular Topology: Molecular Topological Index = 4268
Molecular Topology: Num Rotatable Bonds = 2 Bond(s)
Molecular Topology: Polar Surface Area = 60.69 Å^2
Molecular Topology: Radius = 5 Atom(s)
Molecular Topology: Shape Attribute = 15.0588235294118
Molecular Topology: Shape Coefficient = 1
Molecular Topology: Sum Of Degrees = 36
Molecular Topology: Sum Of Valence Degrees = 62
Molecular Topology: Topological Diameter = 10 Bond(s)
Molecular Topology: Total Connectivity = 0.00283505757266572
Molecular Topology: Total Valence Connectivity = 1.99227538385155 E-05
Molecular Topology: Wiener Index = 582

Dihydroresveratrol (DHRSV)

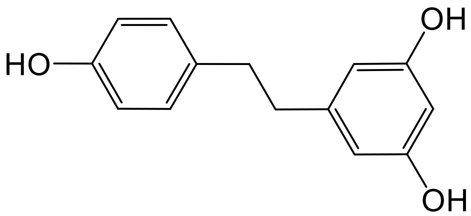

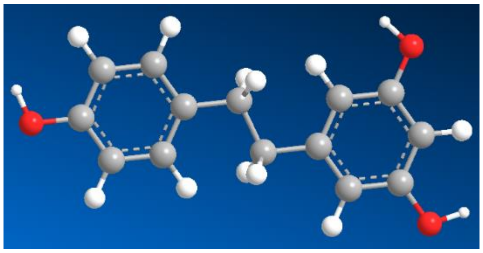

ChemPropStd: Formal Charge = 0
ChemPropStd: Connolly Accessible Area = 437.763 $Å^2$
ChemPropStd: Connolly Molecular Area = 225.595 $Å^2$
ChemPropStd: Connolly Solvent Excluded Volume = 187.027 $Å^3$
ChemPropStd: Exact Mass = 230.0942943157 g/Mol
ChemPropStd: Mass = 230.26300000155
ChemPropStd: Mol Weight = 230.26300000155
ChemPropStd: Number of HBond Acceptors = 3
ChemPropStd: Number of HBond Donors = 3
ChemPropStd: Ovality = 1.42640375564467
ChemPropStd: Principal Moment = 368.843 2971.769 3334.002
ChemPropStd: Elemental Analysis = C, 73.03; H, 6.13; O, 20.84
ChemPropStd: m/z = 230.09 (100.0%), 231.10 (15.1%), 232.10 (1.1%)
ChemPropStd: Mol Formula = C14H14O3
ChemPropStd: Mol Formula HTML = C14H14O3
CLogP Driver: Mol Refractivity = 6.58669996261597
CLogP Driver: Partition Coefficient = 2.58699989318848
Molecular Networks: LogP = 2.31877 Log Units
Molecular Networks: LogS = -2.9915 Log Units
Molecular Networks: pKa = pKa1: 9.54972, pKa2: 9.68852, pKa3: 9.68852 Log Units
Molecular Topology: Balaban Index = 117735
Molecular Topology: Cluster Count = 17
Molecular Topology: Molecular Topological Index = 4268
Molecular Topology: Num Rotatable Bonds = 3 Bond(s)
Molecular Topology: Polar Surface Area = 60.69 $Å^2$
Molecular Topology: Radius = 5 Atom(s)
Molecular Topology: Shape Attribute = 15.0588235294118
Molecular Topology: Shape Coefficient = 1
Molecular Topology: Sum Of Degrees = 36
Molecular Topology: Sum Of Valence Degrees = 60
Molecular Topology: Topological Diameter = 10 Bond(s)
Molecular Topology: Total Connectivity = 0.00283505757266572
Molecular Topology: Total Valence Connectivity = 2.98841307577733 E-05
Molecular Topology: Wiener Index = 582

Piceatannol (PICE)

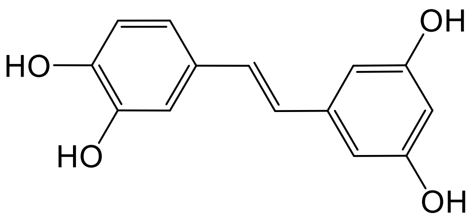

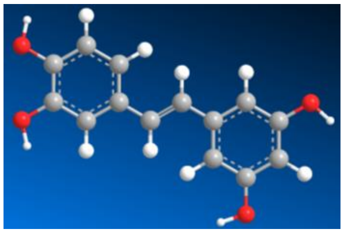

ChemPropStd: Formal Charge = 0
ChemPropStd: Connolly Accessible Area = 434.802 Å^2
ChemPropStd: Connolly Molecular Area = 223.926 Å^2
ChemPropStd: Connolly Solvent Excluded Volume = 180.704 Å^3
ChemPropStd: Exact Mass = 244.0735588736 g/Mol
ChemPropStd: Mass = 244.2460000015
ChemPropStd: Mol Weight = 244.2460000015
ChemPropStd: Number of HBond Acceptors = 4
ChemPropStd: Number of HBond Donors = 4
ChemPropStd: Ovality = 1.4486891934211
ChemPropStd: Principal Moment = 425.765 3184.629 3610.393
ChemPropStd: Elemental Analysis = C, 68.85; H, 4.95; O, 26.20
ChemPropStd: m/z = 244.07 (100.0%), 245.08 (15.1%), 246.08 (1.1%)
ChemPropStd: Mol Formula = C14H12O4
ChemPropStd: Mol Formula HTML = C14H12O4
CLogP Driver: Mol Refractivity = 6.86639976501465
CLogP Driver: Partition Coefficient = 2.23600006103516
Molecular Networks: LogP = 1.7855 Log Units
Molecular Networks: LogS = -2.9088 Log Units
Molecular Networks: pKa = pKa1: 8.60035, pKa2: 9.18246, pKa3: 9.18246, pKa4:14.8832 Log Units
Molecular Topology: Balaban Index = 152012
Molecular Topology: Cluster Count = 18
Molecular Topology: Molecular Topological Index = 4772
Molecular Topology: Num Rotatable Bonds = 2 Bond(s)
Molecular Topology: Polar Surface Area = 80.92 Å^2
Molecular Topology: Radius = 5 Atom(s)
Molecular Topology: Shape Attribute = 16.0555555555556
Molecular Topology: Shape Coefficient = 1
Molecular Topology: Sum Of Degrees = 38
Molecular Topology: Sum Of Valence Degrees = 68
Molecular Topology: Topological Diameter = 10 Bond(s)
Molecular Topology: Total Connectivity = 0.00231481481481481
Molecular Topology: Total Valence Connectivity = 7.71604938271605 E-06
Molecular Topology: Wiener Index = 672

Oxyresveratrol (Oxy-RSV)

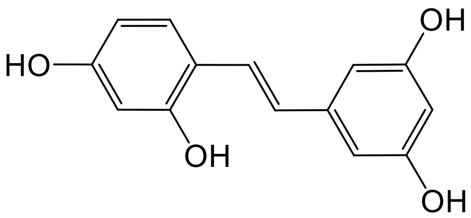

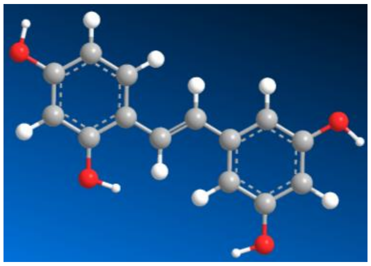

ChemPropStd: Formal Charge = 0
ChemPropStd: Connolly Accessible Area = 429.541 Å^2
ChemPropStd: Connolly Molecular Area = 221.59 Å^2
ChemPropStd: Connolly Solvent Excluded Volume = 179.48 Å^3
ChemPropStd: Exact Mass = 244.0735588736 g/Mol
ChemPropStd: Mass = 244.2460000015
ChemPropStd: Mol Weight = 244.2460000015
ChemPropStd: Number of HBond Acceptors = 4
ChemPropStd: Number of HBond Donors = 4
ChemPropStd: Ovality = 1.44008676511875
ChemPropStd: Principal Moment = 459.116 2893.857 3352.973
ChemPropStd: Elemental Analysis = C, 68.85; H, 4.95; O, 26.20
ChemPropStd: m/z = 244.07 (100.0%), 245.08 (15.1%), 246.08 (1.1%)
ChemPropStd: Mol Formula = C14H12O4
ChemPropStd: Mol Formula HTML = C14H12O4
CLogP Driver: Mol Refractivity = 6.86639976501465
CLogP Driver: Partition Coefficient = 2.16600012779236
Molecular Networks: LogP = 1.13957 Log Units
Molecular Networks: LogS = -2.50538 Log Units
Molecular Networks: pKa = pKa1:8.73176, pKa2: 9.95855, pKa3: 9.95855, pKa4: 12.9427 Log Units
Molecular Topology: Balaban Index = 149955
Molecular Topology: Cluster Count = 18
Molecular Topology: Molecular Topological Index = 4720
Molecular Topology: Num Rotatable Bonds = 2 Bond(s)
Molecular Topology: Polar Surface Area = 80.92 Å^2
Molecular Topology: Radius = 5 Atom(s)
Molecular Topology: Shape Attribute = 16.0555555555556
Molecular Topology: Shape Coefficient = 1
Molecular Topology: Sum Of Degrees = 38
Molecular Topology: Sum Of Valence Degrees = 68
Molecular Topology: Topological Diameter = 10 Bond(s)
Molecular Topology: Total Connectivity = 0.00231481481481481
Molecular Topology: Total Valence Connectivity = 7.71604938271605 E-06
Molecular Topology: Wiener Index = 663

trans-Pterostilbene (PTERO)

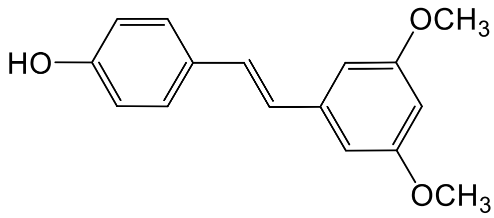

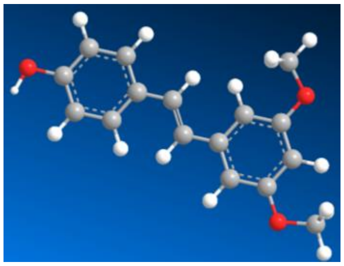

ChemPropStd: Formal Charge = 0
ChemPropStd: Connolly Accessible Area = 480.648 $Å^2$
ChemPropStd: Connolly Molecular Area = 249.942 $Å^2$
ChemPropStd: Connolly Solvent Excluded Volume = 204.911 $Å^3$
ChemPropStd: Exact Mass = 256.1099443799 g/Mol
ChemPropStd: Mass = 256.30100000175
ChemPropStd: Mol Weight = 256.30100000175
ChemPropStd: Number of HBond Acceptors = 3
ChemPropStd: Number of HBond Donors = 1
ChemPropStd: Ovality = 1.48700216559027
ChemPropStd: Principal Moment = 579.501 3384.993 3957.884
ChemPropStd: Elemental Analysis = C, 74.98; H, 6.29; O, 18.73
ChemPropStd: m/z = 256.11 (100.0%), 257.11 (17.3%), 258.12 (1.4%)
ChemPropStd: Mol Formula = C16H16O3
ChemPropStd: Mol Formula HTML = C16H16O3
CLogP Driver: Mol Refractivity = 7.64089965820313
CLogP Driver: Partition Coefficient = 4.17500019073486
Molecular Networks: LogP = 4.04849 Log Units
Molecular Networks: LogS = -4.13642 Log Units
Molecular Networks: pKa = pKa1: 9.50818 Log Units
Molecular Topology: Balaban Index = 197745
Molecular Topology: Cluster Count = 19
Molecular Topology: Molecular Topological Index = 5860
Molecular Topology: Num Rotatable Bonds = 4 Bond(s)
Molecular Topology: Polar Surface Area = 38.69 $Å^2$
Molecular Topology: Radius = 6 Atom(s)
Molecular Topology: Shape Attribute = 17.0526315789474
Molecular Topology: Shape Coefficient = 0
Molecular Topology: Sum Of Degrees = 40
Molecular Topology: Sum Of Valence Degrees = 66
Molecular Topology: Topological Diameter = 11 Bond(s)
Molecular Topology: Total Connectivity = 0.00141752878633286
Molecular Topology: Total Valence Connectivity = 1.66022948654296 E-05
Molecular Topology: Wiener Index = 788

Rotated component matrix

	Components		
	1	2	3
Caco 2_IC50 72h	.110	.119	.808
Caco 2_IC50 48 h	-.129	-.094	.854
Conn. Acc. Area	.959	-.043	.257
Conn. Mol. Area	.955	.012	.273
Conn. Sol. Ex. Vol.	.873	-.028	.454
Exact Mass	.888	.454	-.009
Mass	.888	.454	-.009
Mol. Weight	.888	.454	-.009
HBond Acceptors	.641	.761	-.070
Ovality	.956	.123	-.240
pKa1	-.244	-.779	.420
Mol. Refractivity	.974	.031	-.158
Partition Coeff.	-.101	-.978	-.066
LogP	-.187	-.962	-.091
LogS	-.005	.937	.263
Balaban Index	.936	.299	-.120
Mol. Topol. Index	.960	.223	-.122
Polar Surface Area	.367	.887	-.189
Shape Attribute	.893	.407	-.139
Sum Of Valence Deg.	.743	.618	-.240
Total Connectivity	-.911	-.359	.143
Total Valence Conn.	-.633	-.571	.391
Wiener Index	.930	.343	-.041

Caco-2

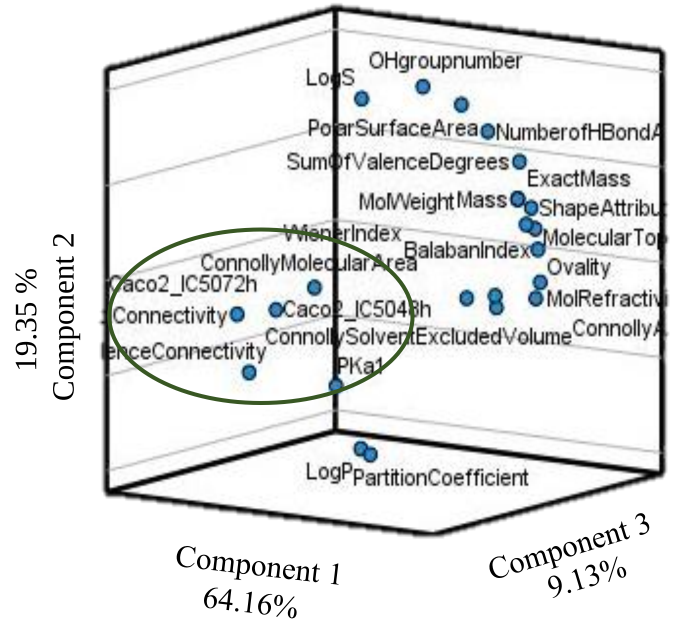

Significant variables in component 3 (when ≥ +0.5)

Figure S5. Principal component analysis in 3-dimensional rotated space for Caco-2 cells. Variables close to significance (0.5) are shown in the rotated component matrix (component 3).
